# *Celine*, a long interspersed nuclear element retrotransposon, colonizes in the centromeres of poplar chromosomes

**DOI:** 10.1093/plphys/kiae214

**Published:** 2024-04-23

**Authors:** Haoyang Xin, Yiduo Wang, Wenli Zhang, Yu Bao, Pavel Neumann, Yihang Ning, Tao Zhang, Yufeng Wu, Ning Jiang, Jiming Jiang, Mengli Xi

**Affiliations:** State Key Laboratory of Tree Genetics and Breeding/Co-Innovation Center for Sustainable Forestry in Southern China, Nanjing Forestry University, Nanjing 210037, China; Department of Plant Biology, Michigan State University, East Lansing, MI 48824, USA; The State Key Laboratory of Crop Genetics and Germplasm Enhancement/Jiangsu Collaborative Innovation Center for Modern Crop Production, Nanjing Agricultural University, Nanjing 210095, China; The State Key Laboratory of Crop Genetics and Germplasm Enhancement/Jiangsu Collaborative Innovation Center for Modern Crop Production, Nanjing Agricultural University, Nanjing 210095, China; Jiangsu Key Laboratory of Crop Genetics and Physiology/Jiangsu Key Laboratory of Crop Genomics and Molecular Breeding/Co-Innovation Centre for Modern Production Technology of Grain Crops/Key Laboratory of Plant Functional Genomics of the Ministry of Education, Yangzhou University, Yangzhou 225009, China; Biology Centre, Czech Academy of Sciences, Institute of Plant Molecular Biology, České Budějovice 37005, Czech Republic; State Key Laboratory of Tree Genetics and Breeding/Co-Innovation Center for Sustainable Forestry in Southern China, Nanjing Forestry University, Nanjing 210037, China; Jiangsu Key Laboratory of Crop Genetics and Physiology/Jiangsu Key Laboratory of Crop Genomics and Molecular Breeding/Co-Innovation Centre for Modern Production Technology of Grain Crops/Key Laboratory of Plant Functional Genomics of the Ministry of Education, Yangzhou University, Yangzhou 225009, China; The State Key Laboratory of Crop Genetics and Germplasm Enhancement/Jiangsu Collaborative Innovation Center for Modern Crop Production, Nanjing Agricultural University, Nanjing 210095, China; Department of Horticulture, Michigan State University, East Lansing, MI 48824, USA; Michigan State University AgBioResearch, East Lansing, MI 48824, USA; Department of Plant Biology, Michigan State University, East Lansing, MI 48824, USA; Department of Horticulture, Michigan State University, East Lansing, MI 48824, USA; Michigan State University AgBioResearch, East Lansing, MI 48824, USA; State Key Laboratory of Tree Genetics and Breeding/Co-Innovation Center for Sustainable Forestry in Southern China, Nanjing Forestry University, Nanjing 210037, China

## Abstract

Centromeres in most multicellular eukaryotes are composed of long arrays of repetitive DNA sequences. Interestingly, several transposable elements, including the well-known long terminal repeat centromeric retrotransposon of maize (CRM), were found to be enriched in functional centromeres marked by the centromeric histone H3 (CENH3). Here, we report a centromeric long interspersed nuclear element (LINE), *Celine*, in *Populus* species. *Celine* has colonized preferentially in the CENH3-associated chromatin of every poplar chromosome, with 84% of the *Celine* elements localized in the CENH3-binding domains. In contrast, only 51% of the CRM elements were bound to CENH3 domains in *Populus trichocarpa*. These results suggest different centromere targeting mechanisms employed by *Celin*e and CRM elements. Nevertheless, the high target specificity seems to be detrimental to further amplification of the *Celine* elements, leading to a shorter life span and patchy distribution among plant species compared with the CRM elements. Using a phylogenetically guided approach, we were able to identify *Celine*-like LINE elements in tea plant (*Camellia sinensis*) and green ash tree (*Fraxinus pennsylvanica*). The centromeric localization of these *Celine*-like LINEs was confirmed in both species. We demonstrate that the centromere targeting property of *Celine*-like LINEs is of primitive origin and has been conserved among distantly related plant species.

## Teaser

A long interspersed nuclear element retrotransposon colonizes centromeric histone H3-associated functional centromeres in *Populus* species.

## Introduction

The centromere was first recognized as the “primary constriction” of metaphase chromosomes and represents the most distinct cytological domain of metaphase chromosomes in higher eukaryotes. Chromatin in the centromeres is defined by the presence of the centromeric histone H3 (CENH3), a centromere-specific H3 histone variant (Henikoff et al. 2001). In most multicellular eukaryotes, centromeres are composed of highly repetitive DNA sequences. Long arrays of satellite repeats and retrotransposons are 2 of the most common types of centromeric repeats (Henikoff et al. 2001; Jiang et al. 2003). The evolutionary dynamics of centromeric satellite repeats have been studied in a number of plant and animal species. Like other satellite repeats, centromeric satellite repeats often evolve rapidly and can be diverged among closely related species (Gong et al. 2012; Zhang et al. 2014; Robledillo et al. 2020). However, certain types of satellite repeats appear to specifically fit in the centromeric chromatin environment. For example, the monomeric units of many classical centromeric satellite repeats are 150–200 bp long, a characteristic length for wrapping a single nucleosome. This 1 repeat–1 CENH3 nucleosome relationship was demonstrated in humans (Hasson et al. 2013) and several plant species (Zhang et al. 2013; Yang et al. 2018; Su et al. 2019). The 155 bp centromeric satellite repeat CentO in rice (*Oryza sativa*) shows both translational and rotational phasing on CENH3 nucleosomes, a feature that may play a role in the stability of centromeric nucleosomes and chromatin (Zhang et al. 2013).

Retrotransposons fall into 2 large groups including long terminal repeat (LTR) and non-LTR elements (Kumar and Bennetzen 1999). A Ty3-*gypsy* type of centromeric LTR retrotransposon (CR) was first discovered in grass species (Aragon-Alcaide et al. 1996; Jiang et al. 1996; Miller et al. 1998; Presting et al. 1998). CR elements were best characterized in rice (CRR, CR of rice) (Cheng, Buell, et al. 2002; Cheng, Dong, et al. 2002; Nagaki et al. 2004; Nagaki et al. 2005) and maize (CRM, CR of maize) (Zhong et al. 2002; Jin et al. 2004; Wolfgruber et al. 2009). Both CRR and CRM elements are highly enriched with CENH3 nucleosomes (Zhong et al. 2002; Nagaki et al. 2005). Cytologically, both CRR and CRM appeared to be largely restricted within the primary constriction of metaphase chromosomes (Cheng, Buell, et al. 2002; Cheng, Dong, et al. 2002; Zhong et al. 2002). Based on phylogeny, CR elements belong to a specific lineage of chromoviruses (Chromoviridae), which has been commonly named as CRM, after the CR of maize (Gorinsek et al. 2004; Kordis 2005). Although CRM lineage elements were found in a wide range of distantly related species of spermatophyta, their centromeric localization was confirmed only in angiosperm species (Neumann et al. 2011; Neumann et al. 2019). A distinctive feature of the CRM elements is the presence of an integrase chromodomain, which differs in sequence from that of other chromoviruses and was hypothesized to be responsible for targeting centromeres (Kordis 2005; Neumann et al. 2019). However, the actual mechanism behind the centromeric specificity of the CRM elements in plants remains a mystery.

In addition to the CRM elements discovered in plants, several other transposable elements (TEs) were found to reside in centromeres. The K111 provirus, a human endogenous retrovirus (HERV), has at least 100 copies in the human genome and is spread across the centromeres of 15 human chromosomes. Chromatin immunoprecipitation (ChIP) experiments confirmed the enrichment of K111 sequences in CENP-A-associated chromatin (Contreras-Galindo et al. 2013). Long interspersed nuclear elements (LINEs) are a group of non-LTR retrotransposons. *LINE-1* (*L1*) represents one of the most abundant retrotransposons in mammalian species, including humans (Beck et al. 2011). Interestingly, the *L1* elements were found to be enriched in the centromeres of phyllostomid bats (de Sotero-Caio et al. 2017). Similarly, LINEs were reported to be in centromeres in banana (D'Hont et al. 2012) and sunflower (Nagaki et al. 2015). Here, we report the discovery of *Celine*, a LINE element that has colonized in the CENH3-associated functional centromeres of poplar chromosomes. On the basis of genome-wide CENH3-binding mapping in *Populus trichocarpa* and pachytene chromosome and DNA fiber-based high-resolution cytological mapping in *Populus simonii*, we were able to analyze the structure, organization, and evolution of a centromeric LINE element with an unprecedented scale and details. The underlying mechanism of *Celine* evolution is discussed.

## Results

### Pt45, a centromeric repeat related to a LINE element in poplar

**Figure 1.**
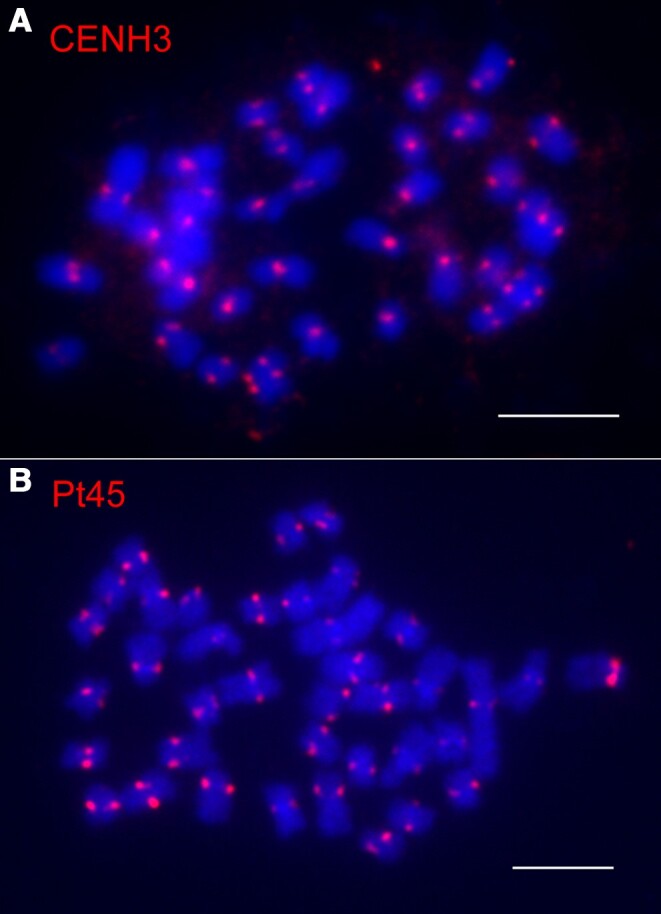
Identification of a centromeric repeat Pt45 in poplar. **A)** Immunofluorescence assay of the anti-CENH3 antibody on somatic metaphase chromosomes prepared from *P. trichocarpa*. **B)** FISH of the Pt45 repeat on the somatic metaphase chromosomes prepared from *P. trichocarpa*. Bars = 5 *μ*m.

To identify DNA sequences associated with the centromeres of poplar chromosomes, we developed an antibody against histone CENH3 of poplar (see Materials and methods). The specificity of this antibody to CENH3 was confirmed by immunofluorescence assay on somatic metaphase chromosomes prepared from *P. trichocarpa* (Fig. 1A). We conducted ChIP using chromatin isolated from young leaf tissue of *P. trichocarpa*. Two DNA libraries, prepared from ChIPed DNA and input DNA, respectively, were prepared and sequenced. We obtained 36.1 and 36.5 million (M) of sequence reads from the 2 libraries. We used 5 M of random reads from the input library to computationally identify repeat sequence clusters using a similarity-based sequence clustering approach (Novak et al. 2010). The proportion (%) of each repeat cluster in the poplar genome was estimated based on the number of reads associated with each cluster. We then mapped the ChIP-sequencing (ChIP-seq) reads to the repeat clusters to identify candidate centromeric repeats based on the enrichment of each repeat cluster in the centromeres (Gong et al. 2012; Yang et al. 2018). We analyzed the top 9 most centromere-enriched repeat clusters (Supplementary Table S1). Five of the repeats were found to be related to the CRM family. Two other repeats were related to the *Athila* and *Tekay* classes of retrotransposons (Supplementary Table S1). Interestingly, a 2,816 bp repeat cluster, Pt45, which exhibited 29 times enrichment in the centromeres and accounted for 0.15% of the poplar genome, showed 99.7% sequence similarity with a LINE-like repeat (L1-1_PTr) that was previously reported in poplar (Jurka 2010). Fluorescence in situ hybridization (FISH) analysis confirmed that Pt45 is located in the centromeres of every *P. trichocarpa* chromosome (Fig. 1B).

### *Celine*, the most abundant LINE family in poplar

**Figure 2.**
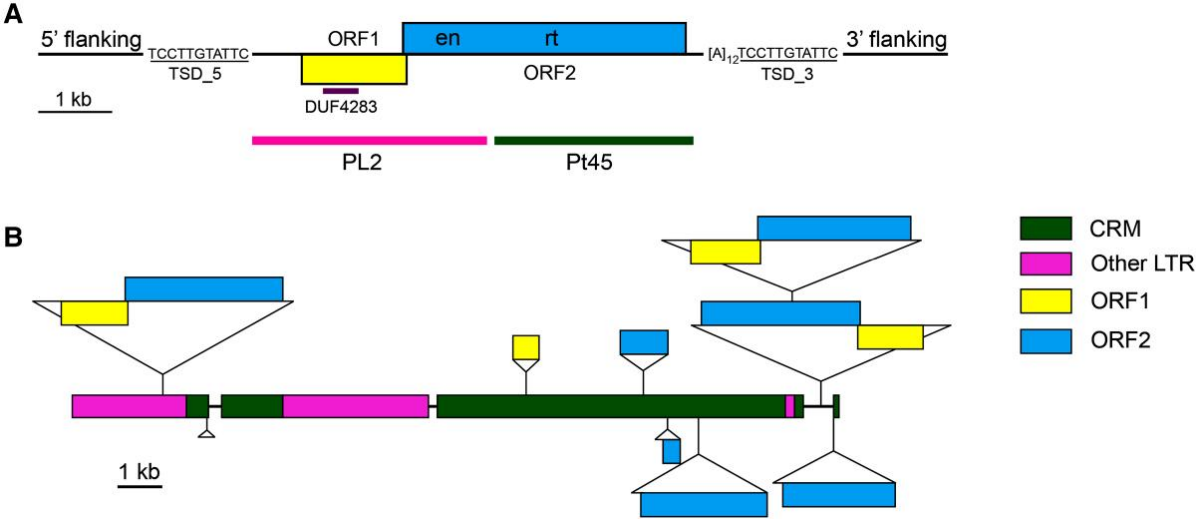
Structure and organization of *Celine* elements. **A)** Structure of a full-length *Celine* element from *P. trichocarpa*. This element is located on chromosome 11 (10,103,867 to 10,109,980 bp, minus strand, element ID Celine_full034). ORF1 contains the DUF4283 domain conserved among LINEs from diverse plant species. ORF2 contains the endonuclease (en) and RT domains. Underlined sequences represent TSD. The 12 bp ploy[A] tail is depicted as [A]_12._ The positions of the 2 FISH probes are indicated. **B)** The organization of *Celine* elements in a single Nanopore read from the *P. simonii* genome. The *Celine* elements are depicted as triangles with coding and noncoding sequences as diagramed in **A)**.

We used Pt45 as an anchor sequence and identified a full-length LINE family, named *Celine* (Centromeric LINE), in the *P. trichocarpa* genome. An example of a full-length *Celine* element is 6,114 bp in length and encodes 2 overlapping open reading frames (ORFs) of 1,617 and 3,765 bp, respectively. The ORFs are preceded by a 635 bp untranslated region (UTR) at the 5′ end and an 189 bp UTR at the 3′ end and terminated by a poly(A) tail of 12 bases (Fig. 2A). The function of the protein encoded by ORF1 is unknown, but its sequence possesses a domain DUF4283 (https://www.ncbi.nlm.nih.gov/Structure/cdd/PF14111) (Fig. 2A) that is conserved among LINEs from diverse plant species, suggesting its importance for LINE replication and/or transposition. ORF2 encodes the domains typical for all autonomous LINEs: an endonuclease and a reverse transcriptase (RT). Given that the transcripts are not spliced, the presence of the 2 ORFs suggests that *Celine* may use noncanonical strategies to translate both ORFs from a single transcript (Gupta and Bansal 2020). Translation reinitiation and an internal ribosome entry site may mediate the initiation of translation, which was described for the *L1* elements in human and mouse (Alisch et al. 2006; Li et al. 2006).

In addition to *Celine*, additional LINE families were identified in the *P. trichocarpa* genome. Using protein sequences from known LINE elements, we retrieved a total of 18 LINE families of all LINEs from *P. trichocarpa*, including *Celine* and 2 families from the Repbase database (Supplementary Table S2). DNA sequences associated with these 18 LINE families account for only 0.85% of the genome. Non-LTR retrotransposons (mainly LINEs) usually occupy a small portion of the plant genomes, but they amplify to a moderate degree in some species. In a previous study, we collected 87 plant genomes with an estimated fraction of non-LTR retrotransposons (Cerbin et al. 2022). Among them, 11 (13%) harbor over 6% of non-LTR elements (range from 6% to 22%), and 17 (20%) contain <0.85%. As a result, the amplification of LINE elements in poplar is limited compared with the majority of other plants. *Celine* is the most abundant LINE element in the *P. trichocarpa* genome (Supplementary Table S2).

### *Celine* colonized in poplar centromeres

**Figure 3.**
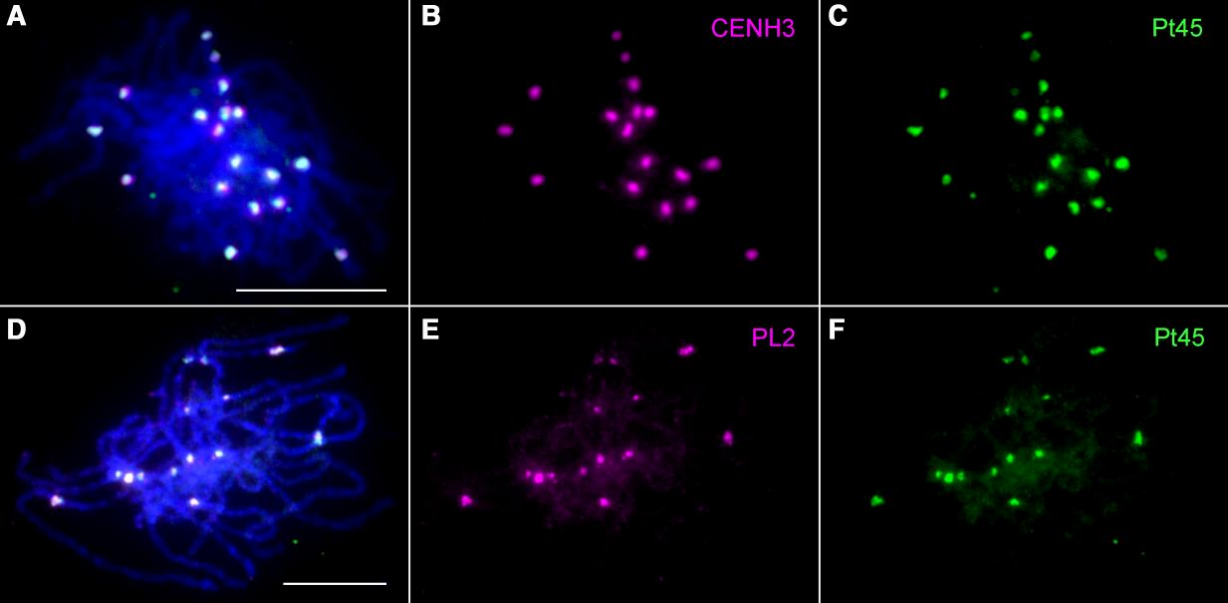
Mapping of CENH3 and *Celine* on pachytene chromosomes of *P. simonii*. **A)** Combined immunofluorescence assay of CENH3 and FISH using Pt45. **B)** Immunofluorescence signals that were digitally separated from **A)**. **C)** FISH signals that were digitally separated from **A)**. **D)** FISH mapping of PL2 and Pt45. **E)** FISH signals of PL2 that were digitally separated from **D)**. **F)** FISH signals of Pt45 that were digitally separated from **D)**. Bars = 10 *μ*m.

To further characterize the location of *Celine* elements in poplar centromeres, we conducted immunofluorescence using the anti-CENH3 antibody (Fig. 3B) followed by FISH using Pt45 (Fig. 3C) on meiotic pachytene chromosomes prepared from *P. simonii*, which diverged from *P. trichocarpa* ∼4.36 million years ago (Mya) (Wu et al. 2020). Pachytene chromosomes have superior cytological resolution compared with the small and highly condensed mitotic metaphase chromosomes of poplar (Xin et al. 2018). The immunofluorescence signals nearly completely overlapped with the FISH signals (Fig. 3A), suggesting that the Pt45 sequence is highly enriched in the CENH3-associated functional centromeres. We observed a similar size and intensity of the immunofluorescence signals in different centromeres (Fig. 3B), suggesting a similar size of the centromeres from different chromosomes. However, the size and intensity of the FISH signals varied significantly among different chromosomes (Fig. 3C), suggesting variable copy numbers of *Celine* in different centromeres.

To further confirm the centromeric localization of the *Celine* elements, we conducted pachytene FISH using another *Celine*-related DNA probe PL2, a 3,334 bp DNA fragment that is immediately adjacent to Pt45 within the full-length *Celine* element (Fig. 2A). The FISH signals generated from Pt45 and PL2 overlapped completely and were confined in the centromeres (Fig. 3, D to F).

### Organization of the *Celine* elements in centromeres

We mapped the 36.1 M CENH3 ChIP-seq reads to the *P. trichocarpa* reference genome (see Materials and methods). The distribution of unique ChIP-seq reads was displayed in 1 kb windows along the 19 poplar chromosomes. Significant sequence enrichment was observed in the centromeres of most poplar chromosomes, except for *Cen13* and *Cen14* (Supplementary Fig. S1). The sizes of the 17 CENH3-binding domains averaged 633 kb, ranging from 427 to 1,267 kb among the 17 chromosomes (Supplementary Table S3). The centromeres of chromosomes 13 and 14 are likely composed of highly repetitive satellite repeats, which may not be included in the current reference genome. A similar phenomenon was previously reported in potato centromeres (Gong et al. 2012). To validate this hypothesis, we performed FISH analysis of all top 9 most abundant repeats identified in the *P. trichocarpa* centromeres (Supplementary Table S1). Interestingly, we discovered that repeat Pt7 hybridized to the 4 centromeres of chromosomes 4 and 13. Repeat Pt20 hybridized to 3 centromeres of both copies of chromosomes 14 and 1 copy of chromosome 5 (Supplementary Fig. S2).

**Figure 4.**
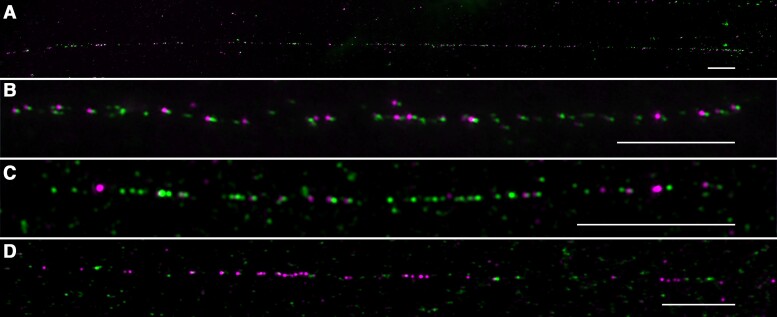
Dual-color fiber-FISH analysis of *Celine* using probe Pt45 (green) and PL2 (magenta) on DNA fibers prepared from *P. simonii*. **A)** A 273.6 *μ*m fiber-FISH signal, which represents ∼878 kb of DNA and likely represents an intact centromere. **B)** A representative fiber-FISH signal with adjacent green and magenta signal dots. **C)** A representative fiber-FISH signal with contiguous green signal dots. **D)** A representative fiber-FISH signal with contiguous magenta signal dots. Bars = 10 *μ*m.

We conducted dual-color FISH on DNA fibers prepared from *P. simonii* using Pt45 (green) and PL2 (magenta) as probes. The 2 probes generated long contiguous fiber-FISH signals (Fig. 4A). We collected many long fiber-FISH signals and selected 12 high-quality signals for measurement (Supplementary Fig. S3). These signals appeared to be intact and spanned 215.1 ± 63 *μ*m (*n* = 12), representing an average of 690.5 ± 202.2 kb using a 3.21 kb/*μ*m conversion rate (Cheng, Buell, et al. 2002). Thus, the total amount of centromeric sequences from the 19 *P. simonii* chromosomes was estimated to be 13.11 ± 3.8 Mb. A significant proportion of the fiber-FISH signals were composed of adjacent green and magenta dots (Fig. 4B), indicating that these *Celine* elements contain both sequences and are likely full-length or near full-length elements. However, we observed contiguous green (Fig. 4C) or magenta signals (Fig. 4D), which were as long as 25.2 *μ*m (∼81 kb). The *Celine* elements associated with these single-color signals are likely truncated. These clustered single-color signals were possibly derived from nested insertions or from regional duplication/amplification events.

### Recent amplification and short life span of *Celine* elements

**Figure 5.**
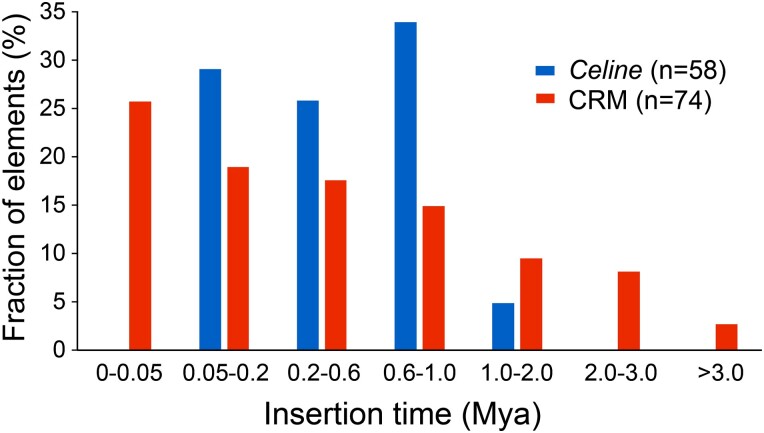
Estimated insertion time of full-length *Celine* elements and intact centromeric retrotransposon of maize (CRM) elements in *P. trichocarpa*. The *x* axes show the insertion time in Mya.

TEs can be grouped into autonomous elements, which code the proteins required for transposition, or nonautonomous elements, which rely on their cognate autonomous elements for movement within the genome. A total of 58 full-length or nearly full-length *Celine* elements (<100 bp truncation at the 5′ end and <500 bp internal deletion) were identified in the latest assembly of the *P. trichocarpa* genome (Pop_tri_v4) (Supplementary Data Set 1 and Supplementary Fig. S4). Among the full-length elements, 14 (24%) harbor both intact ORF1 and ORF2 (Supplementary Data Set 1 and Supplementary Fig. S4), suggesting that they have the potential to encode the functional transposition machinery. Among them, 12 overlap with the CENH3-binding domains, and the 2 additional elements are located within 20 kb of the CENH3-binding domains. As a result, virtually all the putative autonomous elements are buried in the functional centromeres. For the remainder (44) of the elements, the ORFs are disrupted by insertions, deletions, or point mutations (Supplementary Fig. S4). Thus, these elements are likely nonautonomous despite their sizes. The overall nucleotide level pairwise identity among these elements ranged from 93.8% to 99.7%. Because each individual insertion was derived from its immediate ancestral copy, an approximate distribution of elements over time can be estimated through the highest pairwise similarity of elements in the genome. Using an “all versus all match,” the highest pairwise identity for each element varied from 95.2% to 99.7%. This analysis revealed the presence of many recent elements and a few old elements with a median identity of 99.1%. Assuming a mutation rate of *μ* = 1.3 × 10^–8^ per bp per year (Ma and Bennetzen 2004), 55 out of the 58 (95%) of the *Celine* elements were inserted into the genome within 1 million years, with the 3 other elements inserted within 1 to 2 million years, suggesting a recent amplification of the *Celine* family (Fig. 5). Nevertheless, we did not identify 2 identical full-length elements, suggesting a lack of current or extremely recent transposition activity.

As a comparison, we identified 74 intact CRM elements from the *P. trichocarpa* genome and used the sequence identity of the 2 LTRs to estimate the insertion time as described (SanMiguel et al. 1998). The median LTR identity is 99.2%, which is slightly higher than the pairwise identity of *Celine* elements (99.1%). However, the CRM elements and *Celine* elements have distinct amplification spectra (Fig. 5). The oldest CRM element was inserted 3.3 Mya, which is close to the detection limit. Meanwhile, 14 CRM elements have identical LTRs, suggesting current or very recent activity. Among them, only 6 are located in the CENH3-binding domains or within 20 kb flanking regions. The other 8 elements are located on chromosomal arms, with 5 of them harboring intact ORFs. These results suggest that CRM elements have been active from the trackable past. In other words, CRM elements have a much longer life span than the *Celine* elements.

### Targeting specificity of *Celine*

**Figure 6.**
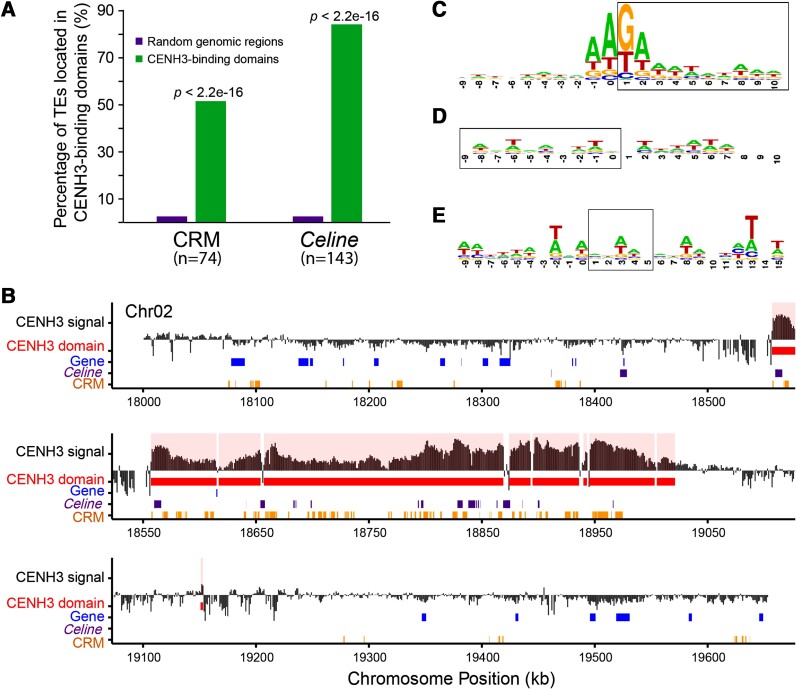
Distribution and targeting specificity of *Celine* and centromeric retrotransposon of maize (CRM) elements. **A)** Percentages of CRM and *Celine* elements located in the CENH3-binding domains. We computationally generated 100 times of random genomic regions that is equivalent to the length of total CENH3-binding domains. The average percentage of CRM and *Celine* within with the random genomic regions is shown in the *y* axis. **B)** Distribution of CRM and *Celine* elements in the centromere of pericentromeric region of chromosome 2 of *P. trichocarpa*. The *x* axes show the chromosome position. CENH3 signal track in the *y* axes indicates the relative enrichment of CENH3 ChIP-seq reads. The CENH3-binding domains are highlighted. Genes, CRM, and *Celine* elements are shown in different tracks. Note: *Celine* elements are rare outside of the CENH3-binding domains. **C)** A pictogram illustrating the sequence at the 5′ end junction of *Celine*. **D)** A pictogram illustrating the sequence at the 3′ end junction of *Celine*. **E)** Target sequence of CRM elements. Sequences inside the boxes represent TSD.

Both CRM and *Celine* elements are highly enriched in the CENH3-binding domains. However, CRM elements are also present throughout individual chromosomes (Supplementary Fig. S5). A majority (84%) of the *Celine* elements was detected in the CENH3-binding domains; in contrast, only 51% of the CRM elements were bound to CENH3 domains (Fig. 6A). *Celine* elements outside of centromeres are relatively rare compared with CRM elements (Fig. 6B). Among the 58 full-length *Celine* elements identified in *P. trichocarpa*, 52 inserted into other TEs; most of these insertions were into *Gypsy*-like LTR retrotransposons, including 30 into CRM elements.

In humans, *L1* elements recognize specific target sequences through its endonuclease domain, which generates staggered nicks prior to retrotransposition (Jurka 1997). To investigate whether *Celine* has any sequence specificity, we retrieved target site duplication (TSD) sequences as well as 10 bp sequences flanking the TSDs. We detected TSDs (10 bp or longer) in 43 of the 58 full-length elements (Supplementary Data Set 1) and in 100 truncated *Celine* elements (Supplementary Data Set 2). The length of TSDs ranged from 10 to 30 bp, with an average of 15 bp. The frequent insertions of *Celine* into other elements raise the question whether the amplification of *Celine* is due to the transposition of the target element also carrying *Celine*. If that is the case, one would expect duplicated TSD and flanking sequences among individual *Celine* elements. However, each *Celine* element has a unique TSD sequence (Supplementary Data Sets 1 and 2). Moreover, all of the 14 CRM elements with identical LTRs do not contain *Celine* sequences or other nested insertions, suggesting elements with nested insertions are unlikely competent for further transposition. Those observations indicate that *Celine* was amplified through their own transposition machinery instead of piggybacking on other TEs including CRM elements.

The average GC content of these retrieved sequences is 35.5%, which is slightly higher than the 33.8% GC in *P. trichocarpa* genome. We examined the base occurrence in each individual position. Base bias is most significant around the junction between the 5′ flanking sequence and TSD. The first nucleotide of the TSD (position 1) is G or T (90%); the nucleotide immediately upstream of the TSD (position 0) and the 2nd nucleotide of the TSD (position 2) are mostly A (66% and 58%, respectively), and the 2nd nucleotide upstream of TSD (position −1) is also biased toward A (57% of occurrences) (Fig. 6C). However, there is no significant bias at the 3′ junction site (Fig. 6D). This suggests that *Celine* primarily targets AAGA/AATA or its variants as the 5′ nicking site. In contrast, the target sequences of CRM elements contain a few AT-rich sites (Fig. 6E); hence, the 2 elements have distinct specificity at the sequence level.

For many CRM retrotransposons, a chromodomain (CHDCR) is present at the C-terminus of integrase, and this domain was assumed to direct CRM elements to centromeres (Neumann et al. 2011). Analysis of intact *Celine* sequences using DANTE failed to detect any CRM*-*related domains (Neumann et al. 2019).

### *Celine* elements in *P. simonii*

To further investigate the genomic distribution and organization of *Celine* elements, we examined the presence of *Celine* elements in our recently developed reference genome of *P. simonii*, which was sequenced using Oxford Nanopore long-read methodology. This reference genome contains 413 Mb of sequences and is comprised of 2,814 contigs assembled using wtdbg2 (Ruan and Li 2020). Sequences similar to *Celine* were found in 245 contigs, accounting for 21.1 Mb of the genome. *Celine* elements represent 11% of the genomic sequences in the 245 contigs. However, 10 of the 245 contigs only contain <2% of *Celine*-related sequences, suggesting that these contigs were likely derived from the boundary regions between centromere and pericentromeric region. If these 10 contigs are excluded, the remaining 235 contigs may represent the core centromeric regions containing 15.2 Mb, which is close to the estimation of 13.1 Mb based on fiber-FISH measurements (see above). The *Celine* elements account for ∼15% of the DNA in the 235 contigs. Similar to what is observed in *P. trichocarpa*, most *Celine* elements (∼65%) in *P. simonii* inserted into LTR retrotransposons, and some of them (∼7%) were nested within themselves. For example, a single nanopore read of 46.5 kb was found to harbor 9 *Celine* elements, which accounts for over half of the sequence (Fig. 2B).

### *Celine*-like LINE elements in plants

**Figure 7.**
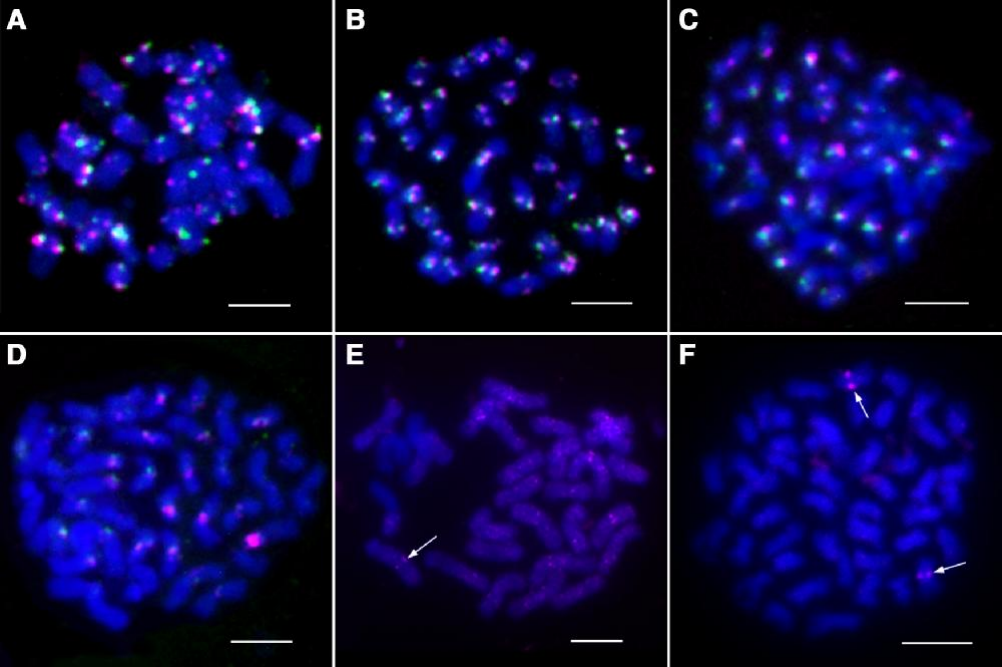
FISH mapping of *Celine* and *Celine*-related LINE elements in different plant species. **A** to **D)** Dual-color FISH of Pt45 (magenta) and PL2 (green) on metaphase chromosomes from *P. deltoides* (A), *P. euphratica* (B), *P. lasiocarpa* (C), and *P. tomentosa* (D). **E)** FISH mapping of a *Celine*-related LINE element in *C. sinensis*. The arrow indicates one of the punctuated centromeric signals. **F)** FISH mapping of a *Celine*-related LINE element in *F. pennsylvanica*. A pair of centromeric signals is indicated by arrows. Bars = 5 *μ*m.

We selected a set of 4 diploid *Populus* species from different sections and a diploid willow species for FISH mapping using Pt45 and PL2. Both probes produced robust centromeric signals on all chromosomes from *Populus deltoides* (section *Aigeiros*), *Populus lasiocarpa* (section *Leucoides*), and *Populus euphratica* (section *Turanga*) (Fig. 7, A to C). However, the FISH signals were significantly weaker on chromosomes of *Populus tomentosa* (section *Leuce*), which diverged from *P. trichocarpa* 13.4 Mya (An et al. 2022) (Fig. 7D). Unambiguous FISH signals were not detected in the centromeres of chromosomes prepared from shrub willow (*Salix suchowensis*), which diverged from poplar 65 Mya (Tuskan et al. 2006).

**Figure 8.**
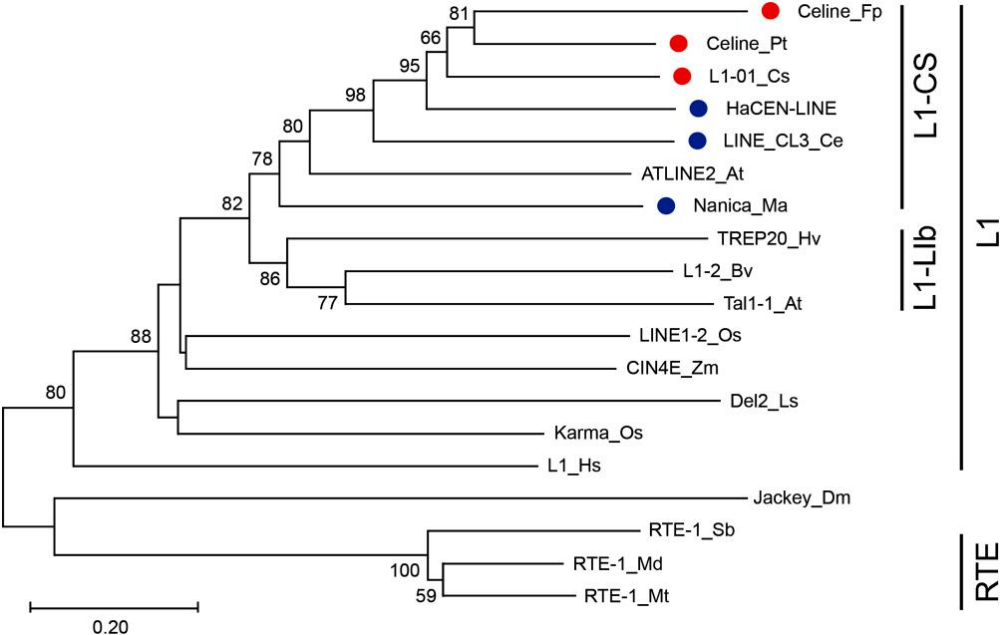
The phylogeny of core RT domain of selected LINE elements. The evolutionary history was inferred using the minimum evolution method with branch length corresponding to the evolutionary distance. Numbers next to the branch indicate the % bootstrap support (1,000 replicates, 50% cutoff). Elements with a red dot are centromere-located elements identified in this study; elements with a blue dot are centromere-located elements identified previously. Abbreviations for species: At, *A. thaliana*; Bv, *Beta vulgaris*; Ce, *C. europaea*; Cs, *C. sinensis*; Dm, *Drosophila melanogaster*; Fp, *F. pennsylvanica*; Ha, *Helianthus annuus*; Hv, *Hordeum vulgare*; Hs, *Homo sapiens*; Ls, *Lilium speciosum*; Ma, *Musa acuminate*; Md, *Malus x domestica*; Mt, *Medicago truncatula*; Os, *O. sativa*; Pt, *P. trichocarpa*; Sb, *Sorghum bicolor*; Zm, *Zea mays*. Abbreviations for elements: L1, human non-LTR retrotransposon LINE-1; L1-Lib, sweet potato LINE LIb; L1-CS, a LINE-like retrotransposon in a sex chromosome of the dioecious plant *Cannabis sativa*; RTE, the RTE-1 (retrotransposable element) element, which was first identified in *Caenorhabditis elegans*.

Previous studies reported 3 LINE elements located in the centromeric regions in plants, including the *Nanica* element in banana (D'Hont et al. 2012; Belser et al. 2021), HaCEN-LINE in sunflower (Nagaki et al. 2015), and LINE-CL3 in the parasitic and holocentric *Cuscuta europaea* (Oliveira et al. 2020; Vondrak et al. 2021). To further test whether *Celine*-like elements are present in additional plant species, we searched for related elements in the National Center for Biotechnology Information (NCBI) nonredundant database and available plant genomes in phytozome (https://phytozome.jgi.doe.gov/). The RT domain from the recovered sequences and those from the 3 known centromeric LINE elements were used to build a phylogenetic tree (Fig. 8). LINEs from plant species fall into 2 clades: the L1 clade and the RTE clade. Of the 7 subclades within the L1 clade (Heitkam et al. 2014), the L1-CS subclade contains LINEs associated with centromeres. We identified 2 putative centromeric LINEs based on their phylogenetic relationship with *Celine* (Fig. 8). The first element, *L1-01_Cs*, was identified in the tea plant (*Camellia sinensis*). FISH analysis using a probe developed from *L1-01_Cs* revealed dispersed signals on most chromosomes. However, punctuated signals were observed in the centromeric regions of several chromosomes (Fig. 7E). The second element, *Cenline_Fp*, was identified in the green ash tree *Fraxinus pennsylvanica*. Distinct centromeric FISH signals were observed on 2 chromosomes (Fig. 7F). Examination of the green ash genome identified 15 *Cenline_Fp* elements on 10 (out of 23) chromosomes, accounting for 0.015% of the genome. Only 4 chromosomes harbor 2 or more elements. The highest pairwise identity of those elements range from 84% to 96% (corresponding to 6.2 to 1.5 million years), suggesting limited recent activity of the *Celine*-like LINE in green ash with much older elements when compared with *P. trichocarpa.*

## Discussion

### The centromere targeting specificity of CRM and *Celine* elements

The centromeric specificity of CRM elements has been one of the most intriguing mysteries in plant centromere biology. The integrase of the CRM elements contains a distinct chromodomain compared with other chromoviruses. This chromodomain has been speculated to play a role in the centromeric specificity of CRM elements (Kordis 2005; Gao et al. 2008). However, extensive analysis of CRM elements from a large number of plant species has yet to reveal a key domain or motif in the integrase that would be required for their centromeric specificity (Neumann et al. 2011). CRM elements intermingle with the centromeric satellite repeat CentO in rice (Cheng, Dong, et al. 2002) and CentC in maize (Jin et al. 2004). Nevertheless, CRM elements do not appear to target satellite repeats in rice and maize. CRM elements become the major centromeric DNA component in plant species lacking a dominant centromeric satellite repeat(s) such as in wheat (Liu et al. 2008; Li et al. 2013).

LINEs have different structures compared with LTR retrotransposons. Proteins encoded by the 2 ORFs of *Celine* do not contain a domain similar to the chromodomain of CRM elements. In general, LINE elements have distinct niches from *Gypsy*-like LTR elements (Cerbin et al. 2022), so it is intriguing to observe that *Celine*-like elements colocalize with CRM elements. The *Nanica* element was extensively intermingled with CRM elements in banana centromeres (Belser et al. 2021). Similarly, *Celine* elements are frequently nested with CRM elements in poplar (see Results). Despite the colocalization of these 2 elements, it is clear they target different sequences (Fig. 6, C to E). Moreover, *Celine* is more specifically located in centromeric regions than CRM elements in poplar (Fig. 6B, Supplementary Fig. S5). As a result, the frequent association of *Celine* with CRM elements is because they are both enriched in centromeric regions, not because they share targeting mechanisms. At this stage, it is unclear how *Celine* targets centromeres. Our analysis indicates it preferentially targets AAGA or AATA sequence motifs. Nevertheless, these combinations of nucleotides are common in the genome. Thus, it is unlikely that these motifs are sufficient to determine its chromosomal locations. It is possible that both *Celine* and CRM elements target a component or different components associated with CENH3 nucleosomes but with different affinities.

### Evolution of *Celine*-like retrotransposons

TEs are major components of eukaryotic genomes. The success of a TE relies on the genetic and epigenetic environments of the genome and the presence of other TE families. TEs are more dynamic and variable than genes due to their ability to amplify and that most of them are dispensable to the host organisms. Most TE families experience a full life cycle of birth, amplification, and extinction (Blumenstiel 2019; Liu et al. 2022). From an evolutionary point of view, individual families of transposons are only transiently present in the genome. LINEs represent the most abundant TE in mammalian genomes (Lander et al. 2001). Whereas in most plant genomes, LINEs only account for a few percent or less (Cerbin et al. 2022). The underlying mechanism for the low abundance of LINEs in plants is not well understood. LINEs were recently found to preferentially insert in introns in sacred lotus (*Nelumbo nucifera*) (Cerbin et al. 2022). Concordantly, the average intron size is 1,988 bp in lotus, which is significantly larger than the average intron sizes in other model plant species, such as *Arabidopsis thaliana* (170 bp) (Kaul et al. 2000) (TAIR10), rice (447 bp) (Kawahara et al. 2013) (IRGSP-1.0), and poplar (<400 bp) (Tuskan et al. 2006). Intriguingly, large introns are a well-known characteristic associated with mammalian genomes. *Celine* is the most abundant LINE element in poplar (Supplementary Table S2). Thus, centromeres may serve as a “safe haven” for *Celine* to survive and thrive, similar to the large introns housing for LINEs in lotus and mammalian genome.

Besides the *Nanica* element from banana (a monocot plant), all the other 5 *Celine*-like elements were found in eudicots. Among them, 4 plants (*C. europaea*, sunflower, tea, and *F. pennsylvanica*) are asterids (but in different orders), whereas *Populus* belongs to rosids (Moore et al. 2010). Interestingly, *Celine* is phylogenetically related to elements from Asterids (Fig. 8). Since asterids diverged from rosids about 125 Mya (Zeng et al. 2017), it suggests that there was either an ancient horizontal transfer event or *Celine* diverged into multiple groups before the divergence of dicots. The presence of *Celine*-like elements in distantly related species supports that *Celine* has an ancient origin. If so, it raises the question of why a *Celine*-like element is absent in most of the sequenced plant genomes while CRM elements are widely present in plants. This is likely attributed to the unique transposition mechanism of LINEs and the high specificity of *Celine*. Upon insertion into the genome, most LTR elements are intact elements, and it is common for an autonomous element to give birth to another autonomous element. In contrast, most LINE elements are truncated at the 5′ end upon insertion, representing nonautonomous elements (Hancks and Kazazian 2016). In this scenario, the consequence of the high specificity of *Celine* leads to the high density of elements in the centromeric regions, with elements nested with each other. This elevates the frequency of truncated elements due to the interruption of the autonomous elements. Even if the element remains intact, the formation of heterochromatin around the centromere may prevent active transcription, resulting in loss of transposition activity and eventual extinction. In contrast, the targeting of CRM elements is not as specific as *Celine*, and multiple putative autonomous elements are found in chromosomal arms, allowing the continuous activity of this family of elements (Fig. 5) and likely contributing to the prevalence of CRM in many plant genomes. Again, this demonstrates the importance of targeting specificity and transposition mode to the success of TEs. From this point of view, the activity of *Celine* is transient on an evolutionary scale, and this explains why it is only detected in a few plant species among thousands of sequenced plant genomes. This model would predict that in a few million years, no FISH signal of *Celine* will be detected in green ash and signals will be detected on approximately half of the poplar chromosomes. As a result, the high centromere specificity of *Celine* represents an evolutionary “dead end.” Meanwhile, new *Celine* elements may evolve from elements with lower centromere specificity or be introduced through horizontal transfer.

## Materials and methods

### Plant materials

Six poplar species (2n = 2x = 38) were used in the present study, including *P. trichocarpa*, *P. simonii*, *P. deltoides*, *P. euphratica*, *P. lasiocarpa*, and *P. tomentosa*. Three additional nonpoplar species were also used for the presence of *Celine*-like elements, including willow (*S. suchowensis*, 2n = 2x = 38), tea plant (*C. sinensis*, 2n = 2x = 30), and green ash tree (*F. pennsylvanica*, 2n = 2x = 46).

### Immunofluorescence assay, FISH and fiber-FISH

A CENH3 antibody was developed as a rabbit polyclonal antiserum and raised against the synthesized peptide of the 20 most N-terminal amino acid sequence (MARTKHPVARKRARSPKRSD) of the CENH3 protein of *P. trichocarpa*. Immunofluorescence was performed according to previously published protocols using the poplar anti-CENH3 antibody (Zhang et al. 2005). For the immunofluorescence combined with FISH assay, after recording of the immunostaining signals, the cytological preparations were washed and followed with a sequential FISH procedure as previously described (Xin et al. 2020).

Preparation of mitotic and meiotic chromosomes was performed according to the protocols described in our previous studies (Xin et al. 2018; Xin et al. 2020). DNA probes specific to the Pt45 and PL2 sequences were amplified via PCR using *P. trichocarpa* DNA as a template. DNA probes of *Celine*-like elements were amplified from the genomic DNA of *C. sinensis* and *F. pennsylvanica* using specific primers (Supplementary Table S4). These amplified DNA fragments were excised from agarose gel, purified, and labeled by nick translation with either digoxigenin-dUTP or biotin-dUTP. FISH and fiber-FISH were performed according to published protocols (Jackson et al. 1998; Xin et al. 2020). Cytological measurements of the fiber-FISH signals were converted into kilobases using a 3.21 kb/*μ*m conversion rate (Cheng, Buell, et al. 2002).

### ChIP-seq and mapping of CENH3-binding domains

ChIP was performed as previously described (Nagaki et al. 2003). Young leaf tissue of *P. trichocarpa* was used to extract chromatin for ChIP assays. Approximately 30 ng of ChIP and input DNA were used for library preparation and sequenced by Illumina HiSeq 2000 platform with 125 bp paired reads. The sequence reads from ChIP and input were mapped to genome v4.0 of *P. trichocarpa* (http://www.phytozome.net/poplar) by Bowtie2 (Langmead et al. 2009). We allowed a 1 bp mismatch threshold between each sequence read and the reference genome. Only the reads mapped to a unique site in the poplar genome were used for further analysis. We divided each poplar chromosome into 1 kb windows and calculated the unique read number per base pair mappable region. Read density was presented as the number of unique reads in a 1 kb window per the length of mappable region in the same window. The final read density was adjusted using the input sequence read data to reduce background signals.

We used SICER2 (Zang et al. 2009) to identify CENH3-binding domains in each poplar centromere within 1 kb windows. We set a mapping stringency that the false discovery rate (FDR) value of a CENH3-subdomain was <0.001 and the fold change of normalized reads number ChIP/input was >5. Identification of centromeric repeats was performed based on the similarity-based clustering method (Novak et al. 2013). Briefly, 5 million reads from the input were used to perform graph-based clustering using the RepeatExplorer web server (https://repeatexplorer-elixir.cerit-sc.cz/). Repeats were identified and classified based on their sequence similarity as individual repeat clusters. To identify repeats enriched in centromeres, ChIP and input reads were mapped to the repeat clusters using BLAT (Kent 2002). The CENH3 enrichment for each repeat was determined as described previously (Gong et al. 2012).

### Identification of *Celine* and CRM elements in the poplar genome and estimation of abundance

To search for full-length *Celine* elements, the initial Pt45 sequence (see Results), which is 2,816 bp in length, was used to search the latest poplar genome assembly ((Tuskan et al. 2006), Pop_tri_v4) using BLASTN (*E* < 10^−10^) (Altschul et al. 1990). Pop_tri_v4 was derived from a 133.2× of PACBIO coverage sequences as well as a high-density poplar map (https://phytozome-next.jgi.doe.gov/info/Ptrichocarpa_v4_1). Sequences matching Pt45 as well as 4 kb flanking sequences on each side were retrieved and aligned with MUSCLE using default parameters (Edgar 2004). The alignment was manually examined for the presence of TSDs flanking the boundary of the alignment. Only TSDs that are 10 bp or longer are considered high confidence and included in Supplementary Data Sets 1 and 2. One of the longest elements, located on chromosome 11 (10,103,867 to 10,109,980 bp; Fig. 2A), harbors 2 apparently intact ORFs when compared with known LINEs in Repbase (Bao et al. 2015) and was considered a representative *Celine* element. Using this element as a standard, *Celine* with a similar length or truncated <100 bp at the 5′ end were considered full-length elements, while other elements were considered truncated elements. For CRM elements, poplar LTR elements in Repbase were extracted. Additional LTR element sequences in poplar were collected using LTR_retriever (Ou and Jiang 2018). All LTR elements were classified using DANTE (Neumann et al. 2019), and those containing CRM domains are considered CRM elements. The sequences of CRM elements and *Celine* were included in a repeat library to mask the poplar genome using RepeatMasker (https://www.repeatmasker.org/), with the abundance of each element assessed based on the length of each element masked. For *Celine* elements, both full-length and 5′ end truncated elements are considered a copy. Fragments (without either end) are not included in copy number estimations.

### Target specificity of *Celine* and CRM elements

For chromosomal level distribution of *Celine* and CRM elements, each chromosome was divided into bins that are 100 kb in length. The fraction of *Celine* or CRM elements was estimated based on the length they covered in each bin. Thereafter, the relative abundance value of the bin with the highest fraction was set to 100 and used to normalize the values in other bins. For the sequence preference at the junction sites of *Celine*, sequences at the 5′ junction site (10 bp upstream of TSD plus 10 bp into TSD) were retrieved and the sequence logo was generated using WebLogo (Crooks et al. 2004). Sequences at the 3′ junction site (the last 10 bp of the TSD plus 10 bp downstream of TSD) were processed similarly. The sequence logo was generated for CRM elements with the 5 bp TSD plus 10 bp flanking sequences on each side. To evaluate the number of *Celine* elements inserted into CRM elements, 150 bp sequence downstream of each *Celine* element was masked using a CRM element library. If the flanking sequence was masked, the relevant *Celine* was considered to be inserted into a CRM element.

### Phylogenetic analysis

The RT domain of *Celine* elements and other LINEs were aligned with MUSCLE using default parameters (Edgar 2004). The origin of sequences used in phylogenetic analysis is listed in Supplementary Tables S5 and S6. The RT domain was defined based on comparison with ORF2 of L1 from the human genome (511 to 773 amino acids, GenBank: AAA51622.1). Phylogenetic trees were generated using a neighbor-joining method with MEGA (Kumar et al. 2018). Support for the internal branches of the phylogeny was assessed using 1,000 bootstrap replicates.

## Supplementary Material

kiae214_Supplementary_Data

## Data Availability

ChIP-seq data sets have been submitted to NCBI under the BioProject accession number PRJNA1021681. The genome assembly of *P. simonii* has been deposited to Genome Warehouse in the National Genomics Data Center (NGDC) under accession number GWHDUDC00000000 (https://ngdc.cncb.ac.cn/gwh/).

## Supplementary data

The following materials are available in the online version of this article.

**Supplementary Figure S1.** Distribution and density of CENH3 ChIP-seq reads on each chromosome of *P. trichocarpa*.

**Supplementary Figure S2.** FISH mapping of centromeric repeats Pt7 and Pt20 in *P. trichocarpa*.

**Supplementary Figure S3.** A set of 12 high-quality long contiguous fiber-FISH signals derived from Pt45 (green) and PL2 (magenta).

**Supplementary Figure S4.** Structure of additional 57 full-length or nearly full-length *Celine* elements in *P. trichocarpa*.

**Supplementary Figure S5.** Distribution and density of CENH3, *Celine*, and CRM on each chromosome of *P. trichocarpa*.

**Supplementary Table S1.** Nine most abundant repeat clusters identified in the centromeres of *P. trichocarpa* chromosomes.

**Supplementary Table S2.** Amplification of LINE elements in *P. trichocarpa*.

**Supplementary Table S3.** Size of CENH3-binding chromosomal domains in *P. trichocarpa*.

**Supplementary Table S4.** PCR primers used in the study.

**Supplementary Table S5.** Elements used in phylogenetic analysis (from NCBI).

**Supplementary Table S6**. Elements used in phylogenetic analysis (from other resources).

**Supplementary Data Set 1.** List of full-length *Celine* elements in *P. trichocarpa*.

**Supplementary Data Set 2.** List of truncated *Celine* elements with TSD in *P. trichocarpa*.

## Dive Curated Terms

The following phenotypic, genotypic, and functional terms are of significance to the work described in this paper:

dna Gramene: EO:0007532
dna Araport: EO:0007532

TES Gramene: AT3G43210
TES Araport: AT3G43210

proteins CHEBI: CHEBI:36080
