# Supplementary material for: Celine, a long interspersed nuclear element retrotransposon, colonizes in the centromeres of poplar chromosomes
Source: Plant Physiol. 2024 Apr 23;195(4):2787–98. doi: 10.1093/plphys/kiae214 (PMC11288735; doi:10.1093/plphys/kiae214)
Supplement: kiae214_Supplementary_Data [file kiae214_supplementary_data.zip › PP2023RA01582R1_Supplementary_Data.pdf]

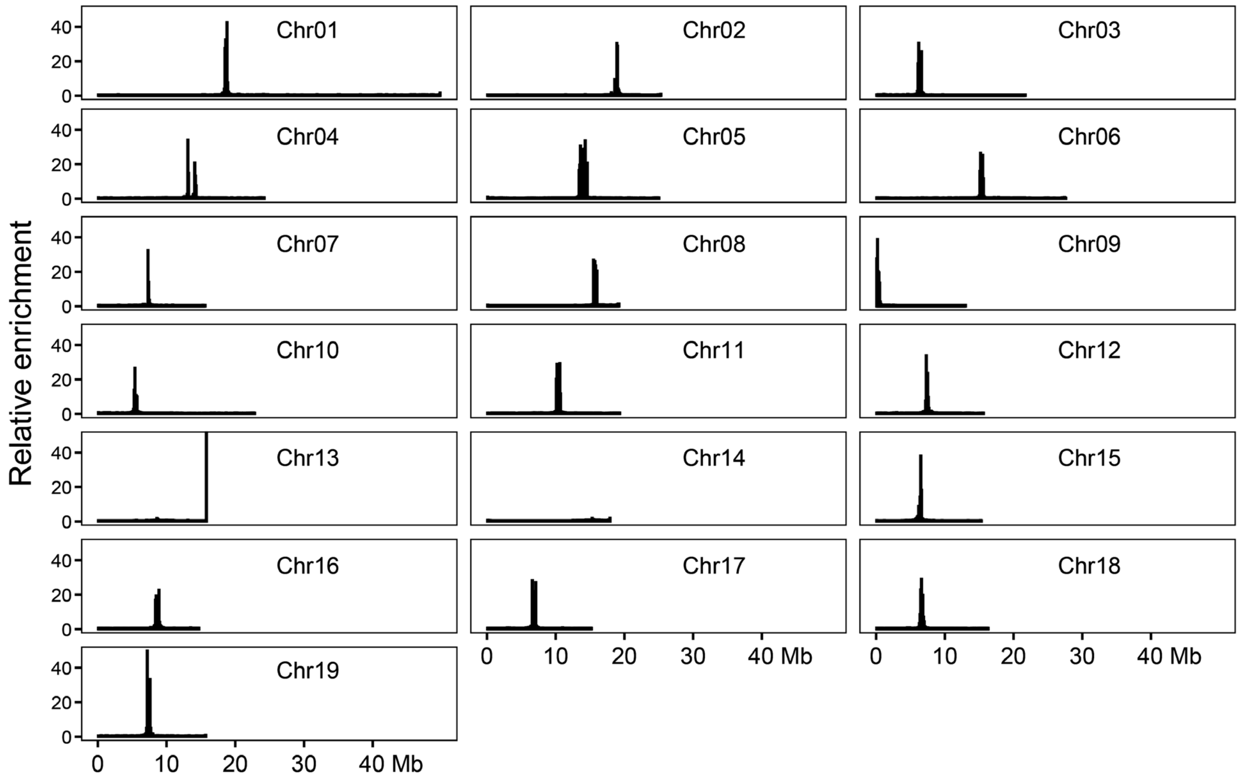

Supplementary Figure S1. Distribution and density of CENH3 ChIP-seq reads on chromosomes of *P. trichocarpa*. The *x* axes show the position on each poplar chromosome. The *y* axes show the chromatin immunoprecipitation-sequencing (ChIP-seq) read density, which is represented by the total number of sequence reads in a 100-kb window per base pair mappable region and adjusted by the input. A major centromeric histone H3 (CENH)-binding domain is observed on all chromosomes, except for chromosomes 13 and 14. Note: Chromosome 13 is metacentric chromosome (Xin et al. 2020). Thus, the enriched sequence reads located at the end of chromosome 13 likely represents mis-assembled sequences derived from the centromere(s) of chromosome 13 and/or other chromosomes.

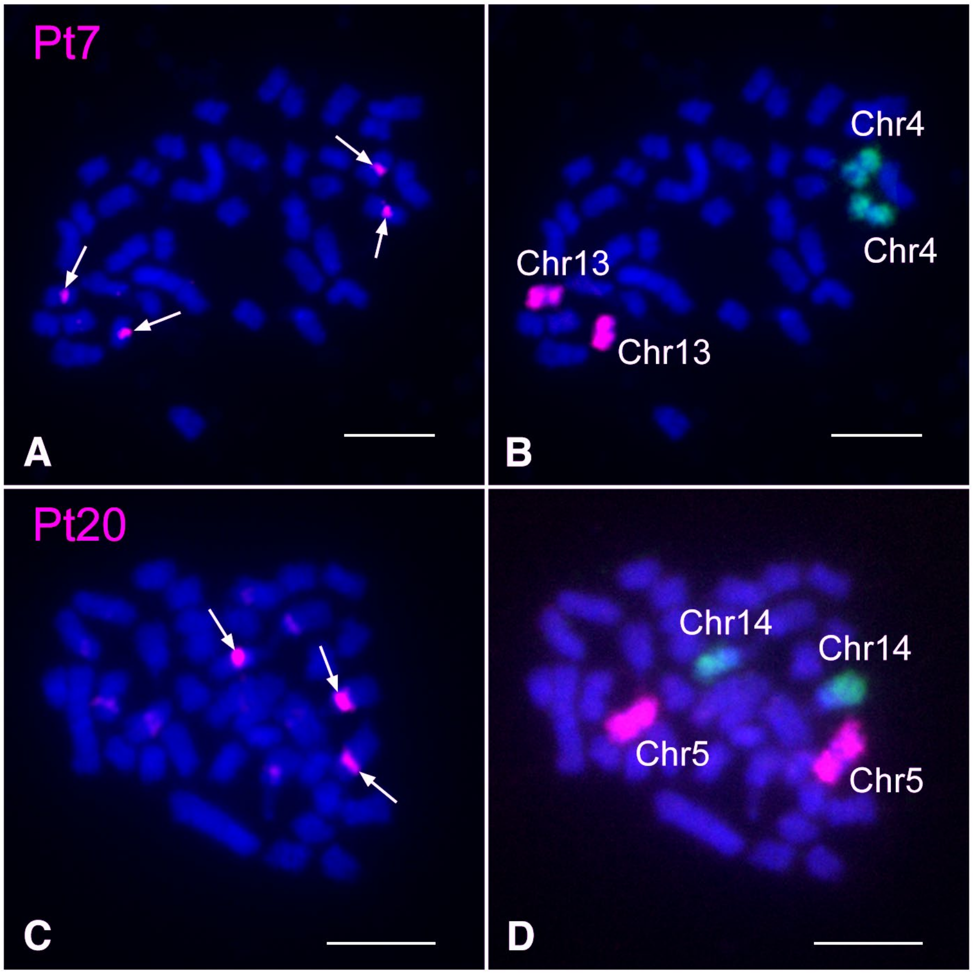

Supplementary Figure S2. FISH mapping of centromeric repeats Pt7 and Pt20 in *P. trichocarpa*. **(A)** FISH mapping of centromeric repeat Pt7. Arrows indicate major signals located in four centromeres. **(B)** Chromosome painting on the same metaphase cell (a) using painting probes specific to chromosome 4 (green) and chromosome 13 (red), respectively. **(C)** FISH mapping of centromeric repeat Pt20. Arrows indicate major signals located in three centromeres. **(D)** Chromosome painting on the same metaphase cell (c) using painting probes specific to chromosome 14 (green) and chromosome 5 (magenta), respectively. Bars = 5 µm.

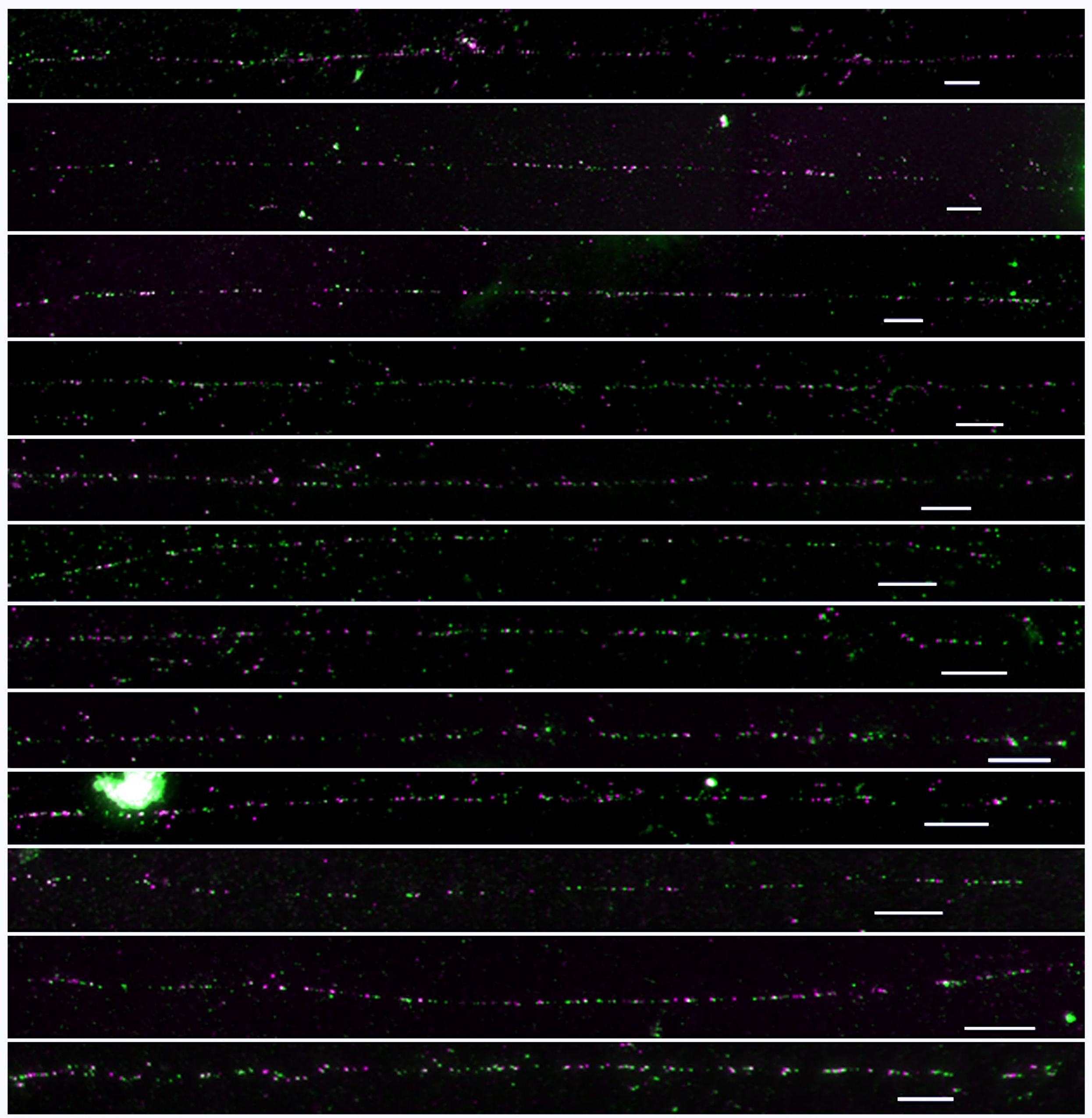

Supplementary Figure S3. A set of 12 high-quality long contiguous fiber-FISH signals derived from Pt45 (green) and PL2 (magenta). The third image from the top is also used as Figure 4A to illustrate signals derived from a complete centromere. Bars = 10 µm.

Supplementary Figure S4_part 1

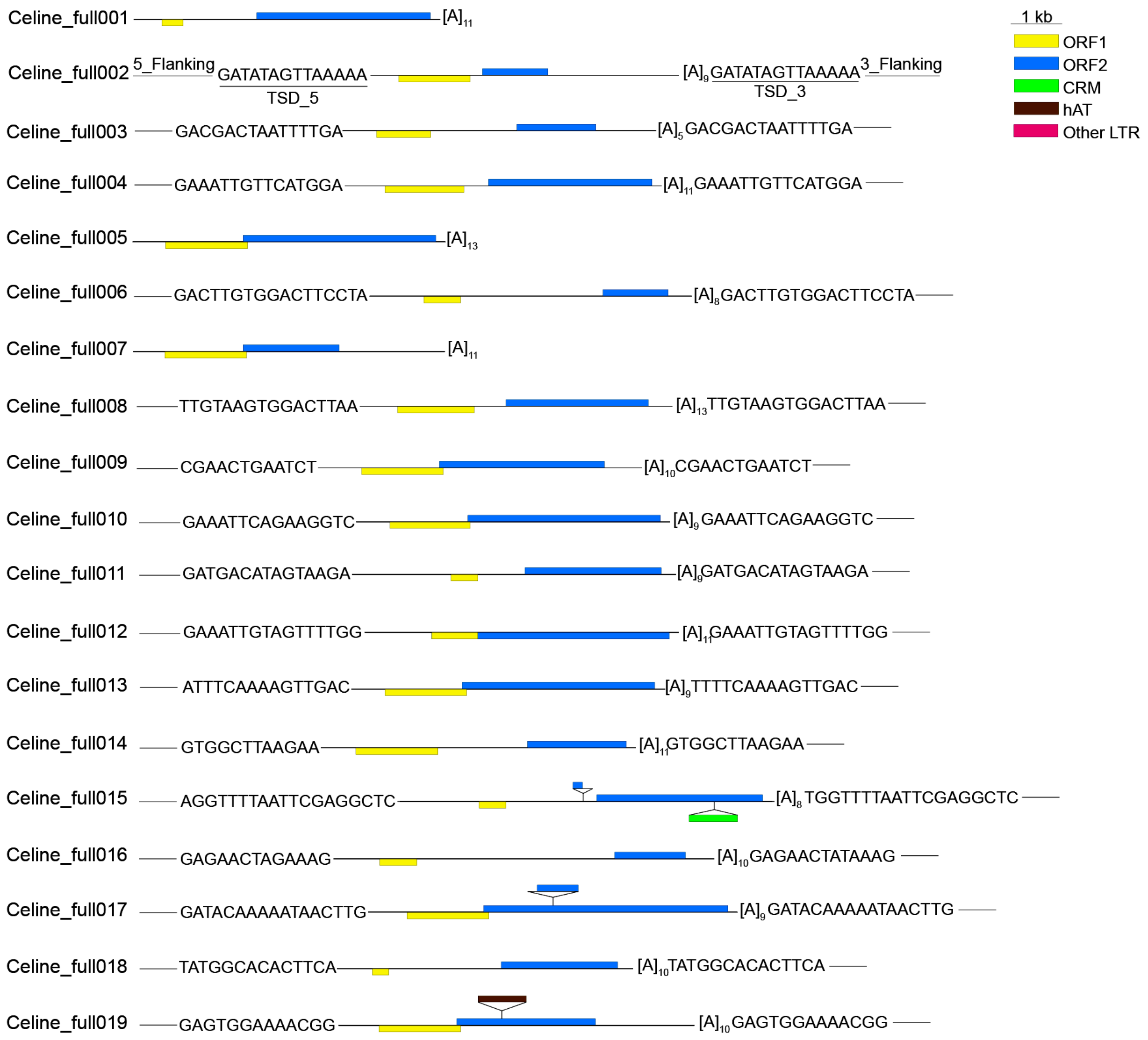

Supplementary Figure S4_part 2

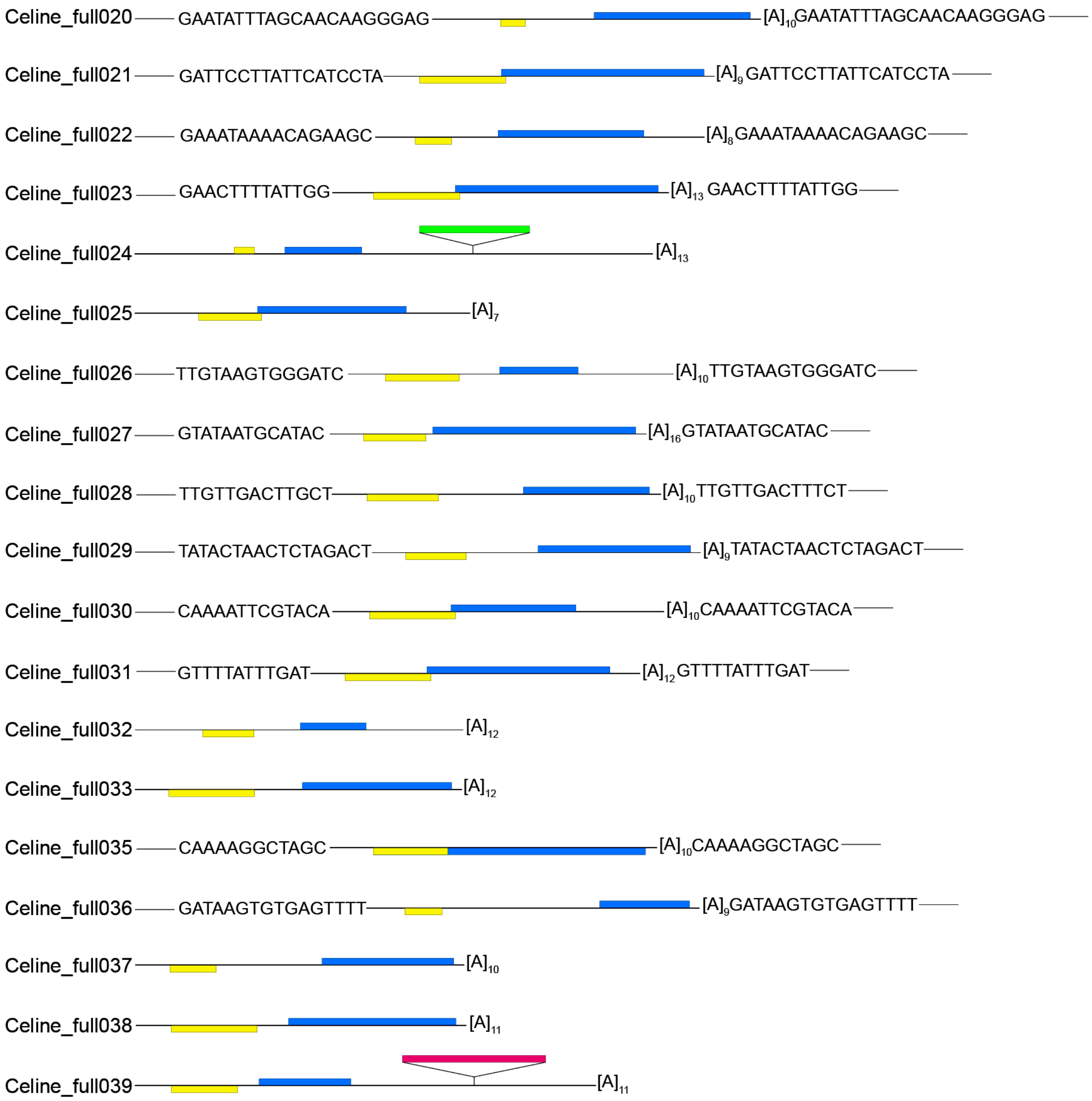

Supplementary Figure S4_part 3

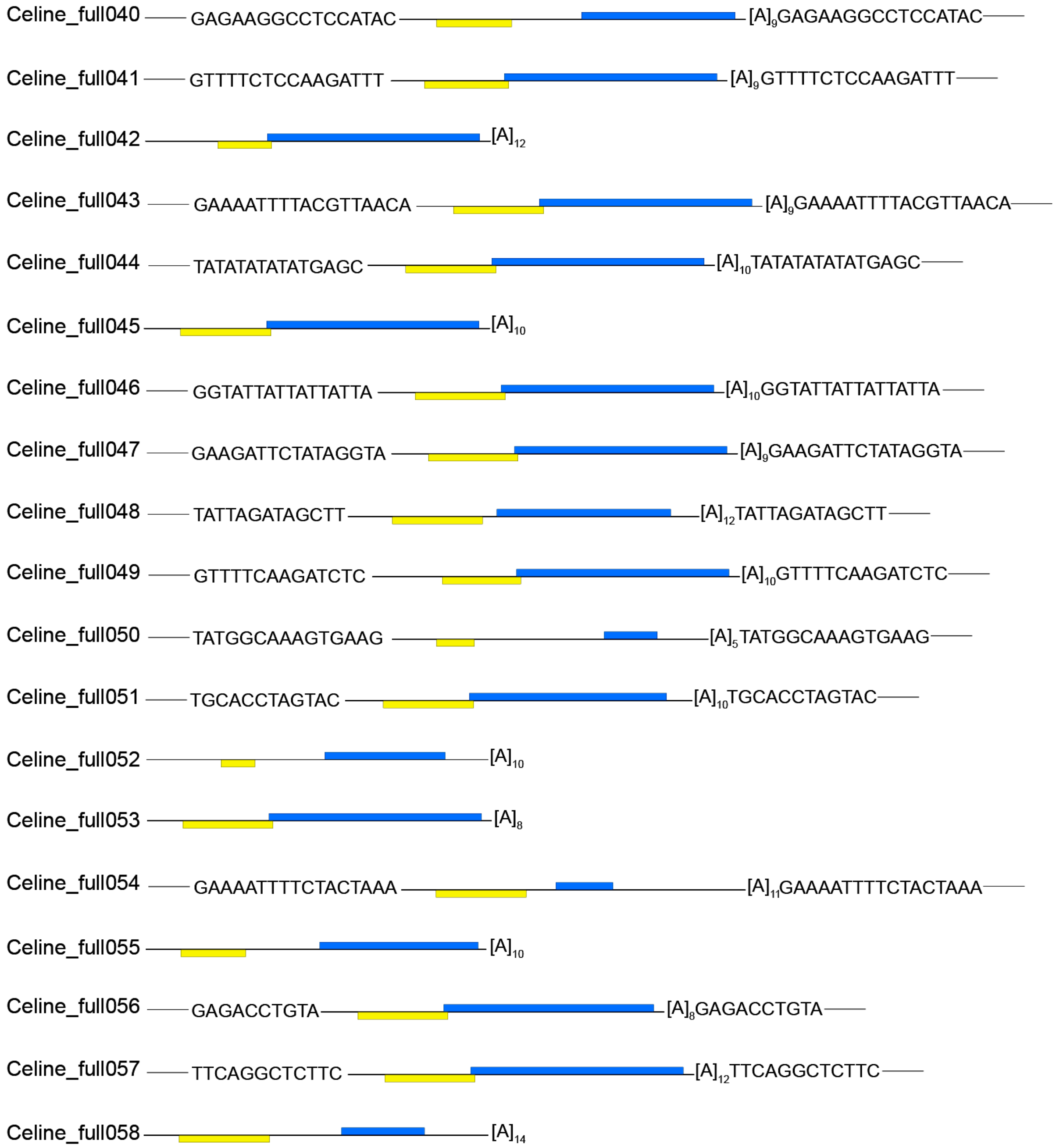

Supplementary Figure S4. Structure of additional 57 full-length or nearly full-length *Celine* elements in *P. trichocarpa*. Each element is depicted in the same way as in Figure 2a. If the open reading frame (ORF) is interrupted by insertion, deletion, frameshift and premature stop code, the largest in-frame ORF is shown. For *Celine*_full012 and *Celine*_full035, there is no frame shift between the two ORFs, so the sequences corresponding to ORF1 (yellow) and ORF2 (blue) in other elements formed a single ORFs within the two elements. If the element contains a nested insertion(s), the structure is predicted based on the putative sequence before the insertion. CRM: centromeric retrotransposon of maize; LTR: long terminal repeat; hAT: a class II transposable element.

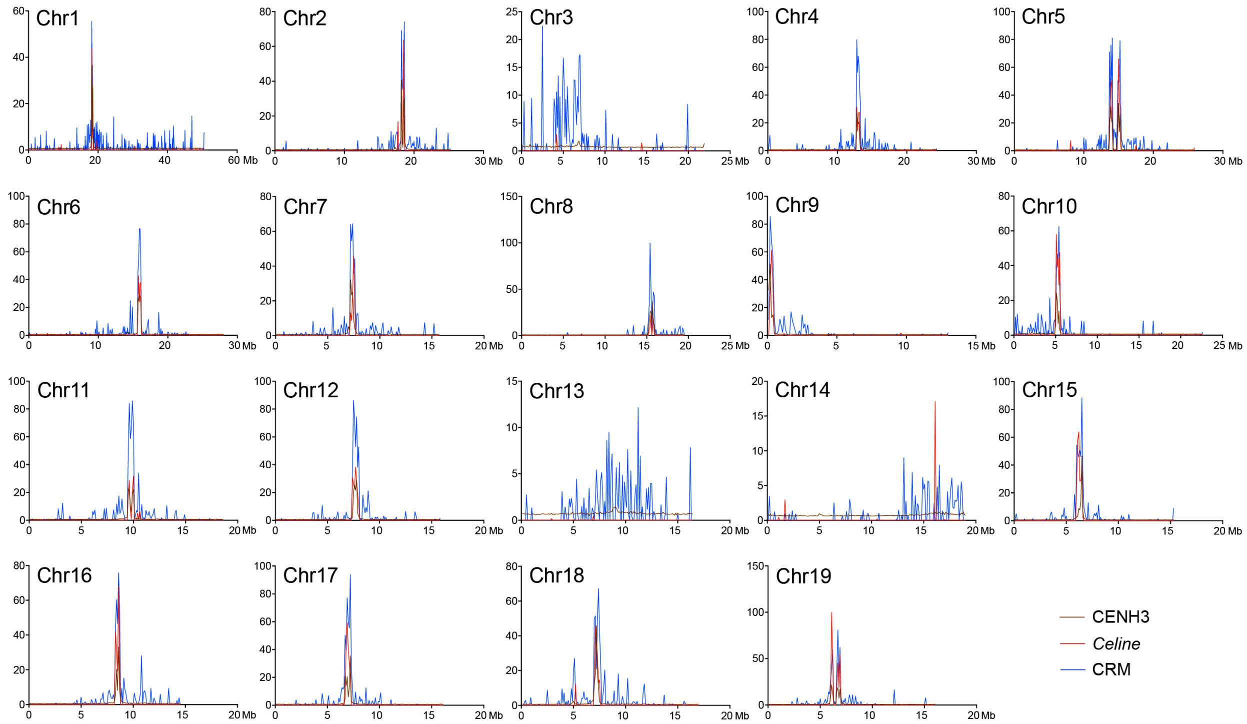

Supplementary Figure S5. Distribution and density of CENH3, *Celine* and CRM on each chromosome of *P. trichocarpa*. The *x*-axes show the physical distance (Mb) on each poplar chromosome with the bin size of 100 kb. The *y*-axes show the relative abundance of centromeric histone H3 (CENH3, dark brown line), *Celine* (red line) and centromeric retrotransposon of maize (CRM, blue line) on each chromosome in 100-kb bins. The relative abundance was calculated by setting the value for the bin with the highest genomic fraction as 100.

Supplementary Table S1. Nine most abundant repeat clusters identified in the centromeres of *P. trichocarpa* chromosomes

Repeat cluster	ChIP/input ratio	Genome proportion (%)	Representative contig	Contig size (bp)	Sequence homology with
CL6	46.5	0.42	Pt6	827	CRM
CL22	39.5	0.22	Pt22	2036	CRM
CL32	29.8	0.19	Pt32	5541	CRM
CL45	28.8	0.15	Pt45	2816	*Celine*
CL19	19.0	0.23	Pt19	1893	CRM
CL20	12.3	0.22	Pt20	1520	Unknown
CL33	10.8	0.19	Pt33	754	CRM
CL14	9.5	0.26	Pt14	483	Athila
CL7	9.3	0.35	Pt7	772	Tekay

Supplementary Table S2. Amplification of LINE elements in *P. trichocarpa*

Element*	Copy number§	Length (bp)	Fraction of the genome (%)
L1-03_Pt	49	86521	0.022
L1-04_Pt	2	12669	0.003
L1-05_Pt	240	454038	0.116
L1-06_Pt	37	132659	0.034
L1-07_Pt	19	29925	0.008
L1-08_Pt	83	242796	0.062
L1-09_Pt	193	104408	0.027
L1-10_Pt	45	164488	0.042
L1-11_Pt	50	82171	0.021
L1-12_Pt	5	16061	0.004
L1-13_Pt_IC	7	35854	0.009
L1-14_Pt	14	24913	0.006
L1-15_Pt_IC	6	10067	0.003
L1-45_Pt (*Celine*)	465	1317191	0.336
L1-51_Pt	25	202795	0.052
L1-52_Pt	35	151375	0.039
L1-2_PTr	171	194319	0.050
L1-4_PTr	59	75803	0.019
Total	1505	3338053	0.853

* L1-2_PTr and L1-4_PTr are from Repbase database, other elements are identified in this study.
§ Copy number including both full-length and truncated copies.

Supplementary Table S3. Size of CENH3-binding chromosomal domains in *P. trichocarpa*

Chr.	CENH3-binding domain				Chr. length	Proportion[a]
	Start (Mb)	End (Mb)	Length (kb)	Total length (kb)	(Mb)	(%)
1	18.37	18.82	456	456	49.79	0.92%
2	18.56	19.15	596	596	25.24	2.36%
3	6.06	6.60	540	540	21.68	2.49%
[b]4-1	12.98	13.18	207	1267	24.14	5.25%
4-2	14.00	14.24	244			
[c]5-1	13.33	13.83	501	1247	24.98	4.99%
5-2	14.08	14.58	493			
6	15.01	15.58	574	574	27.52	2.09%
7	6.84	7.46	618	618	15.56	3.97%
8	15.41	15.95	544	544	19.20	2.83%
9	0.01	0.50	496	496	12.99	3.82%
10	5.22	5.67	454	454	22.80	1.99%
11	10.05	10.67	627	627	19.29	3.25%
12	7.11	7.54	427	427	15.59	2.74%
15	6.08	6.55	472	472	15.23	3.10%
16	8.35	8.94	597	597	14.62	4.08%
17	6.51	7.08	574	574	15.19	3.78%
18	6.26	6.95	688	688	16.26	4.23%
19	7.09	7.67	581	581	15.62	3.72%

[a] Proportion (%) = centromere length/chromosome length X 100.

[b] *Cen4* consists of two CENH3 subdomains (4-1: 207 kb; 4-2: 244 kb) that are separated by a H3 subdomain of 0.82 Mb.

[c] *Cen5* consists of two CENH3 subdomains (5-1: 501 kb; 5-2: 493 kb) that are separated by a H3 subdomain of 0.25 Mb.

Supplementary Table S4. PCR primers used in the study

Repeat	Primer	Sequence
Pt45	Pt45_F	TTAGGGAAACCAATACAACCAG
	Pt45_R	ACGAGGCAGTCAAATCAGAAGT
PL2	PL2_F	CTACATCTACTCCGTCCACC
	PL2_R	TTGCCAATAACCTTTACCAC
L1-01_Cs	L1-01_Cs_F	ATGTTATCCAAGCCATTAG
	L1-01_Cs_R	CAGCCCTCAACTTCGTG
Cenline_Fp	Cenline_Fp_F	CTTCAAGAGGACGGGAATG
	Cenline_Fp_R	TGGAACACCAAGATAGCG

Supplementary Table S5. Elements used in phylogenetic analysis (from NCBI)

Element Name	Species	Accession no
Tal1-1_At	*Arabidopsis thaliana*	AAA75254.1
L1-01_Cs	*Camellia sinensis*	XP_028111743.1
Jockey_Dm	*Drosophila melanogaster*	AAA28675.1
Cenline_Fp	*Fraxinus pennsylvanica*	OU503052.1 (10494961 to 10495758)*
HaCEN-LINE	*Helianthus annuus*	LC075745.1
L1_Hs	*Homo sapiens*	AAA51622.1
Del2_Ls	*Lilium speciosum*	Z17425.1
Nanica_Ma	*Musa acuminate*	AC226048.1 (103776 to 104567)*
Karma_Os	*Oryza sativa*	AB081316.2
Tx1_Xl	*Xenopus laevis*	AAA49976.1

*The relevant accessions contain other sequences in addition to the element, thus, the coordinates are provided

Supplementary Table S6. Elements used in phylogenetic analysis (from other resources)

Element Name	Species	Original name	Database or reference
ATLINE2_At	*Arabidopsis thaliana*	*ATLINE2*	girinst.org/repbase
L1-2_Bv	*Beta vulgaris*	*BvL1-2*	girinst.org/repbase
LINE_CL3_Ce	*Cuscuta europaea*	*LINE-CL3Contig152_1368-2183*	Vondrak et al. 2021
TREP20_Hv	*Hordeum vulgare*	*TREP20*	girinst.org/repbase
RTE-1_Md	*Malus x domestica*	*RTE-1_Mad*	girinst.org/repbase
RTE-1_Mt	*Medicago truncatula*	*RTE1_MT*	girinst.org/repbase
LINE1-2_Os	*Oryza sativa*	*OSLINE1-2*	girinst.org/repbase
RTE-1_Sb	*Sorghum bicolor*	*RTE-1_SBi*	girinst.org/repbase
CIN4E_Zm	*Zea mays*	*CIN4E_ZM*	girinst.org/repbase
