# Supplementary material for: Celine, a long interspersed nuclear element retrotransposon, colonizes in the centromeres of poplar chromosomes
Source: Plant Physiol. 2024 Apr 23;195(4):2787–98. doi: 10.1093/plphys/kiae214 (PMC11288735; doi:10.1093/plphys/kiae214)
Supplement: kiae214_Supplementary_Data [file pp.01582.2023-s04.pdf › pp.01582.2023-s04.pdf]

Plant Physiology®

Celine, a LINE retrotransposon colonized in the centromeres of poplar chromosomes

Jiming Jiang, Haoyang Xin, Yiduo Wang, Wenli Zhang, Pavel Neumann, Yihang Ning, Tao Zhang, Yufeng Wu, Ning Jiang, Mengli Xi, and Yu Bao

Review Timeline:

Submission Date:	10-Oct-2023
Editorial Decision:	22-Nov-2023
Revision Received:	27-Feb-2024
Accepted:	14-Mar-2024

22-Nov-2023

Dr. Jiming Jiang
Michigan State University
East Lansing, Michigan 48824

RE: Celine, a LINE retrotransposon colonized in the centromeres of poplar chromosomes

Dear Dr. Jiang:

We have received reviews of your manuscript, which you may view at the end of this letter. On the basis of the advice received and my own evaluation, I believe that, pending revision, this article may ultimately be acceptable for publication in Plant Physiology.

In particular, please pay more attention to the first reviewer's comments:
1. Centromeric LINE elements have been reported in a variety of plants, including banana, sunflower, parasitic and holocentric Cuscuta europaea, as well as in humans. So the results reported here are not novel. It's not surprising to find diverse repetitive sequences in the centromere region across different species, which is a well-acknowledged phenomenon.
2. The manuscript lacks a detailed exploration into the mechanisms underlying the presence or targeting of LINE transposons. Questions surrounding the accumulation of Celine LINE elements in poplar chromosomes remain unanswered.
3. I found that the P. trichocarpa genome version employed by the authors was published in 2006. The assembly and annotation quality of this version is inadequate for current analytical standards. Many centromere sequences have not been anchored to the chromosome, and many assembly errors were detected in centromere regions. The annotations of Celine elements along with nucleotide level pairwise identity lack precision, potentially rendering some results questionable. Given the advancements in genome sequencing techniques, elucidation of the mechanisms could be significantly enhanced using HiFi and Nanopore sequencing methods.
4. The resolution provided by the mapping of CENH3 and Celine on pachytene or mitotic chromosomes of P. simonii using FISH or Fiber-FISH is insufficient to substantiate the colonization of Celine elements in poplar centromeres and their organizational structure therein.
5. The manuscript mentions a recently developed reference genome of P. simonii, sequenced using the Oxford Nanopore long-read methodology. However, no detailed information or citations were included in this manuscript. Is this data published? If the genome are still under restriction, its presentation in this manuscript is inappropriate.

When you are ready to submit your revision, click the below link and find the red arrow under your Author Tasks heading for this revision submission.

If you want to visit this version of the manuscript, you may click the link below and then click on the folder that reads "Post Decision Manuscripts".

Link Not Available

When uploading the revised version of this article, please be sure to include as complete an answer as possible to the referees' criticisms during the first round of reviews in the "response to reviewers" section.

Plant Physiology considers a revision for up to 60 days after a manuscript has been provisionally accepted; limited extension of this timeframe is at the discretion of the Monitoring Editor. A reminder notice will be sent to you 2 weeks before the deadline, at which time you may choose to contact the editorial office to request an extension; if so, please provide a justification for your request. After the expiration of this deadline, the article would need to be submitted as a resubmission and would be handled as such.

Thank you very much for giving us an opportunity to review this work. I look forward to receiving the next version.

Sincerely,

Plant Physiology **oard of Editors**

Want to add this revision deadline to your calendar? Click below!

-- Reviewer comments:
Reviewer #1 (Comments for the Author):

Xin et al reported a line retrotransposon in the centromere of poplar chromosome. Their study suggested different targeting mechanisms of LINE and CRM in the centromere, and they also compared the Celine signal distributions among close related plant species. These results provide good example for discovering Celine-like LINEs among distantly related plant species. However, there are several concerns with these results. The results of this manuscript are not solid for the conclusion, due to outdated genome version and limited resolution of FISH method. The novelty is not sufficient to provide new insights into centromere evolution as similar results has been reports in other species as well as the authors themselves. The manuscript was piled up with descriptions where it lacked the key mechanism. The specificity for the poplar species has not been introduced, and the stamen of the relationship between these distantly or closely related species is particularly limited. It restricted the reader of the paper.

Major concerns:
1. Centromeric LINE elements have been reported in a variety of plants, including banana, sunflower, parasitic and holocentric Cuscuta europaea, as well as in humans. So the results reported here are not novel. It's not surprising to find diverse repetitive sequences in the centromere region across different species, which is a well-acknowledged phenomenon.

2. The manuscript lacks a detailed exploration into the mechanisms underlying the presence or targeting of LINE transposons. Questions surrounding the accumulation of Celine LINE elements in poplar chromosomes remain unanswered.

3. I found that the P. trichocarpa genome version employed by the authors was published in 2006. The assembly and annotation quality of this version is inadequate for current analytical standards. Many centromere sequences have not been anchored to the chromosome, and many assembly errors were detected in centromere regions. The annotations of Celine elements along with nucleotide level pairwise identity lack precision, potentially rendering some results questionable. Given the advancements in genome sequencing techniques, elucidation of the mechanisms could be significantly enhanced using HiFi and Nanopore sequencing methods.

4. The resolution provided by the mapping of CENH3 and Celine on pachytene or mitotic chromosomes of P. simonii using FISH or Fiber-FISH is insufficient to substantiate the colonization of Celine elements in poplar centromeres and their organizational structure therein.

5. 5. The manuscript mentions a recently developed reference genome of P. simonii, sequenced using the Oxford Nanopore long-read methodology. However, no detailed information or citations were included in this manuscript. Is this data published? If the genome are still under restriction, its presentation in this manuscript is inappropriate.

Minor comments
1. P5 line 126-127. The manuscript lacks detailed information regarding the enriched clusters repeats. Were other repeat clusters such as CRM or satellite repeats identified in poplar chromosome? The author mentioned "These three centromeres (Cen3, Cen13, and Cen14) are likely to be mostly composed of highly repetitive satellite repeats". The highly repetitive satellite repeats needs further elucidation or evidence.

2. P5 line 134 "We used Pt45 as an anchor sequence and identified a full-length LINE family, named Celine (Centromeric LINE)". Why did the author used this full-length LINE as example show in figure 2. The rationale behind showcasing this particular full-length LINE in Figure 2, as opposed to the structures of other 58 full-length or near FL-Celine, is not clear.

3. P5 line 144: (Gupta and Bansal, 2020) (Gupta and Bansal, 2020). a repeated citation

4. P6 line 164. "these 18 LINE families account for only 0.75% of the genome, indicating limited amplification of LINE elements in this genome." It lacks a comparative framework. Given that LINE elements typically occupy small proportions in most plant genomes, a control or a comparative analysis is essential for contextual understanding.

5. The choice of Populus simonii to confirm Celine in poplar centromeres is not adequately justified. The resolution provided by FISH and immunofluorescence signals may not substantiate the occupancy of CENH3 in these LINE sequences. Furthermore, the probes derived from poplar might exhibit deviations in signal intensity, and may not accurately reflect the signal strength differences in Populus due to the considerable variance in centromere sequences among different species.

6. P7. Line 193. "These three centromeres are likely to be mostly composed of highly repetitive satellite repeats." needs further substantiation. No satellite repeats have been identified and reported in the enriched cluster repeats. FISH signals of Pt45 were

detected in all the chromosomes, however, no Celine repeats were identified in the reference genome, while many CRM repeats were assembled in the genome. It's very strange.

7. P7 line 202. "Thus, the total amount of centromeric sequences from the 19 P. simonii chromosomes 203 was estimated to be 13.11 {plus minus} 3.8 Mb." The estimation of centromere size using FISH signals of Pt45 and PL2 may not provide an accurate representation of the core CENH3 region. This method might not be suitable given the current advancements in genome sequencing and assembly.

8. P8. "Celine primarily targets AAGA/AATA or its variants as the 5' nicking site. In contrast, the target sequences of CRM elements contain few AT-rich sites, hence the two elements have distinct specificity at the sequence level." The current genome is published in 2006, and the results may not be accurate. The dissimilar targeting mechanisms between Celine and CRM, being different types of retrotransposons, are not surprising and warrant a more detailed exploration of the underlying mechanisms.

9. P9 line 244. "presence of Celine elements in our recently developed reference genome of P.simonii, which was sequenced using Oxford Nanopore long-read methodology." However, no detailed information or citation were include in this manuscript. If this data is unpublished or if the genome is under restriction, its inclusion in this manuscript is inappropriate.

Reviewer #2 (Comments for the Author):

This manuscript entitled "Celine, a LINE retrotransposon colonized in the centromeres of poplar chromosomes" by Xin et al. investigated a centromeric LINE element, Celine, in Populus species. Overall, the study is neat and straight forward. I have some questions.

1. Figure 2b, lines 217-219 and other places in the paper demonstrate that Celine elements are inserted in CRM LTR retrotransposons. It is not clear to me whether the amplification of Celine is due to its own activity or the byproduct of the amplification of the CRM elements. If the authors compare the ages of these CRM elements in centromeres, what is their amplification time? Is it similar to the divergence time of the Celine elements?

2. I am also curious whether the authors identified any centromeric satellite repeats associated with or near those Celine elements?

3. Abstract, Page 2, line 42, please also add the full name of "CRM" when it is first used.

4. Figure 1b, it seems that the red signal of the rightmost chromosome is not in the middle? Why?

5. Figure 2b, what are the red rectangles?

6. Results, page 6, line 160, please change into "in the P. trichocarpa genome".

7. Results, page 9, line 253-255, "Similar to what is observed in P. trichocarpa, most Celine elements (~65%) in P. simonii inserted into LTR retrotransposons, and some of them (~7%) were nested within themselves." I think it is really hard to tell whether there was one or multiple insertion events. It is largely possible that one or few Celine elements inserted into LTR retrotransposons, which got amplification, resulting in the amplification of the Celine elements. However, from the reading of here, it seems that the authors thought this was due to the multiple insertions. I do not see the data supporting it.

8. Results, page 12, line 356-359, it seems to me that Celine became not active and less important for centromere function anymore.

Response to comments from Reviewer #1

Xin et al reported a line retrotransposon in the centromere of poplar chromosome. Their study suggested different targeting mechanisms of LINE and CRM in the centromere, and they also compared the Celine signal distributions among close related plant species. These results provide good example for discovering Celine-like LINEs among distantly related plant species. However, there are several concerns with these results. The results of this manuscript are not solid for the conclusion, due to outdated genome version and limited resolution of FISH method. The novelty is not sufficient to provide new insights into centromere evolution as similar results has been reports in other species as well as the authors themselves. The manuscript was piled up with descriptions where it lacked the key mechanism. The specificity for the poplar species has not been introduced, and the stamen of the relationship between these distantly or closely related species is particularly limited. It restricted the reader of the paper.

Response: We respect these comments from the Reviewer. We agree that our manuscript does not solve the puzzle of the molecular mechanism of how *Celine* elements target the chromatin domains marked by CENH3 (see our detail response on this below). We have tried to be more focusing on the comparative analysis of *Celine* and CRM elements and explaining the "self-termination" characteristics of *Celine*-like LINEs in eukaryotes.

Major concerns:

1. Centromeric LINE elements have been reported in a variety of plants, including banana, sunflower, parasitic and holocentric Cuscuta europaea, as well as in humans. So the results reported here are not novel. It's not surprising to find diverse repetitive sequences in the centromere region across different species, which is a well-acknowledged phenomenon.

Response: Centromeric repeats often diverge rapidly. Closely related eukaryotic species often have different centromeric repeats. Thus, presence of a phylogenetically related LINE in the centromeres of very few and phylogenetically unrelated eukaryotes is a fascinating biological phenomenon. Although centromeric LINEs were previously reported in a few species, the centromeric LINEs were not fully characterized and analyzed in banana, sunflower, or in humans. We defined the CENH3-binding domains in every poplar chromosome and identified every *Celine* element within or outside a functional centromere, which was not accomplished previously in any other species. The comparative analysis of *Celine* and CRM elements reveals different genomic targeting mechanisms associated with these two fascinating TE families enriched in functional centromeres. Most importantly, our manuscript provides insight explaining why centromeric LINEs exist sporadically in a few distantly related species due to its self-limiting amplification in a highly specialized chromosomal domain. In contrast, CRM is nearly universally present in various plant lineages.

2. The manuscript lacks a detailed exploration into the mechanisms underlying the presence or targeting of LINE transposons. Questions surrounding the accumulation of Celine LINE

elements in poplar chromosomes remain unanswered.

Response: We agree it would be ideal to unveil the mechanistic targeting mechanism of the *Celine* transposons. However, this is not a trivial task, and the targeting mechanism has been revealed only for very few transposable elements. The CRM element has been studied extensively since 1998, yet it is still largely unknown for its centromere targeting mechanism. At this stage, we don't have the resources or tools to conduct the experiments proposed by the reviewer. We did not intend to answer all the questions regarding the accumulation mechanism of *Celine* in centromeres, nor did we claim we do. Having said that, we did make meaningful progress toward understanding the targeting mechanism of *Celine*. For example, we have demonstrated that *Celine* is more specific than CRM in targeting the functional centromeres marked by the centromeric histone CENH3. Moreover, *Celine* has a moderate targeting specificity at the sequence level, which is different from that of CRM. In addition, we demonstrate that CRM has a more persisting activity than *Celine*, likely due to their distinct transposition mechanisms. This may explain why CRM is more prevalent in centromeres than *Celine* elements. We consider those results, together with other findings in this study, are interesting to the readers of Plant Physiology.

3. I found that the P. trichocarpa genome version employed by the authors was published in 2006. The assembly and annotation quality of this version is inadequate for current analytical standards. Many centromere sequences have not been anchored to the chromosome, and many assembly errors were detected in centromere regions. The annotations of Celine elements along with nucleotide level pairwise identity lack precision, potentially rendering some results questionable. Given the advancements in genome sequencing techniques, elucidation of the mechanisms could be significantly enhanced using HiFi and Nanopore sequencing methods.

Response: We appreciate the comments and suggestion. We have re-performed all of our analyses related to the genomic sequence using the latest assembly (v4), which is derived from high coverage of PACBIO sequences as well as a high-density genetic map. With the new assembly, we did retrieve more elements and the average age of elements seems to be slightly younger than that from the previous assembly. However, the new analyses did not change any conclusion. We hope the update is acceptable.

4. The resolution provided by the mapping of CENH3 and Celine on pachytene or mitotic chromosomes of P. simonii using FISH or Fiber-FISH is insufficient to substantiate the colonization of Celine elements in poplar centromeres and their organizational structure therein.

Response: We respect the comments from the Reviewer. In our manuscript, we relied on the positions of the CENH3-binding domains to determine the "centromeric" or "non-centromeric" position of each *Celine* element in *P. trichocarpa*. Pachytene chromosome-based FISH and immunofluorescence with anti-CENH3 antibody allowed us to validate the enrichment of *Celine* elements in the CENH3-binding domains in another poplar species (*P. simonii*) in which ChIP-seq data is not available. The CENH3-binding domain occupies approximately 1/50 of a pachytene chromosome. Thus, pachytene chromosome-based assays provide a very powerful

tool to reveal whether a DNA element is associated and enriched within the functional centromere. Furthermore, our mapping results are consistent with the analyses with genomic sequences.

5. The manuscript mentions a recently developed reference genome of P. simonii, sequenced using the Oxford Nanopore long-read methodology. However, no detailed information or citations were included in this manuscript. Is this data published? If the genome are still under restriction, its presentation in this manuscript is inappropriate.

Response: The reference genome of *P. simonii* has been released and is indicated in the "Data availability" statement of the manuscript: The genome assembly of *P. simonii* has been deposited to Genome Warehouse in The National Genomics Data Center (NGDC) under accession number GWHDUDC00000000 (https://ngdc.cncb.ac.cn/gwh/).

Minor comments

1. P5 line 126-127. The manuscript lacks detailed information regarding the enriched clusters repeats. Were other repeat clusters such as CRM or satellite repeats identified in poplar chromosome? The author mentioned "These three centromeres (Cen3, Cen13, and Cen14) are likely to be mostly composed of highly repetitive satellite repeats". The highly repetitive satellite repeats needs further elucidation or evidence.

Response: We intended to focus on *Celine* in the manuscript. There are many other classes of repetitive DNA elements associated with the centromeres. Most of them were derived or amplified from CRM elements. However, amplification of CRM-related centromeric repeats has been reported in several plant species. Thus, it has limited value for us to describe these repeats again.

To address the concern from the Reviewer, we have added a table (new Table S1) that lists the top 9 most abundant repeats identified in poplar centromeres. Amazingly, two of these repeats (Pt7 and Pt20) hybridized to Cen13 and Cen14, respectively, which confirm our prediction that these two centromeres are likely to be composed of highly repetitive satellite repeats. These results, including a new **Figure S2**, have been added in the revised manuscript. Note: the CENH3-binding domain associated with Cen3 was found in the v4 assembly of the *P. trichocarpa* reference genome.

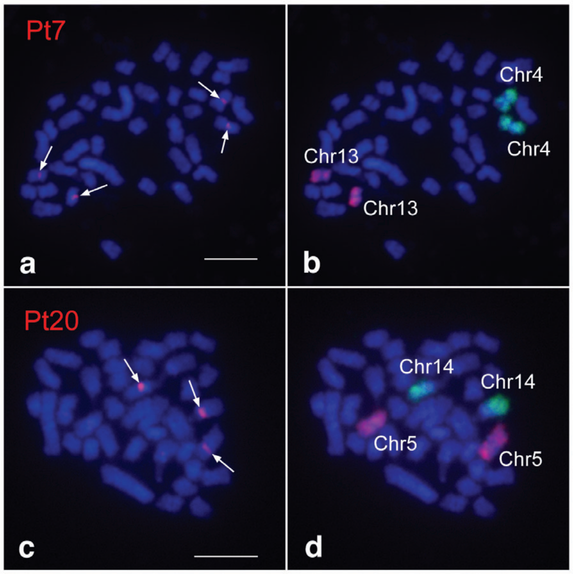

Figure S2. FISH mapping of centromeric repeats Pt7 and Pt20 in *P. trichocarpa*. **(a)** FISH mapping of centromeric repeat Pt7. Arrows indicate major signals located in four centromeres. **(b)** Chromosome painting on the same metaphase cell (a) using painting probes specific to chromosome 4 (green) and chromosome 13 (red), respectively. **(c)** FISH mapping of centromeric repeat Pt20. Arrows indicate major signals located in three centromeres. **(d)** Chromosome painting on the same metaphase cell (c) using painting probes specific to chromosome 14 (green) and chromosome 5 (red), respectively. Bars = 5 µm.

2. P5 line 134 "We used Pt45 as an anchor sequence and identified a full-length LINE family, named Celine (Centromeric LINE)". Why did the author used this full-length LINE as example show in figure 2. The rationale behind showcasing this particular full-length LINE in Figure 2, as opposed to the structures of other 58 full-length or near FL-Celine, is not clear.

Response: We chose this particular full-length LINE as an example because it structurally well represents *Celine*. However, this element disappeared in the newest assembly (v4), so we switched to another element as a representative. Obviously, there is not enough space in one figure to show the structure of all 58 elements with reasonable details. In the revised manuscript, we have carefully examined the structure of all full-length elements and shown the structure of the additional 57 elements in a new supplementary figure (**Figure S4**). We included description about the structure of these elements in the revised manuscript.

3. P5 line 144: (Gupta and Bansal, 2020) (Gupta and Bansal, 2020). a repeated citation

Response: The error is fixed, thanks.

4. P6 line 164. "these 18 LINE families account for only 0.75% of the genome, indicating limited amplification of LINE elements in this genome." It lacks a comparative framework. Given that LINE elements typically occupy small proportions in most plant genomes, a control or a

comparative analysis is essential for contextual understanding.

Response: We appreciate this valuable comment. We had some discussion in the original manuscript, but we have added some background/comparative information alongside this statement. Please see below:

"DNA sequences associated with these 18 LINE families account for only 0.85% of the genome. Non-LTR retrotransposons (mainly LINEs) usually occupy a small portion of the plant genomes, but they amplify to a moderate degree in some species. In a previous study, we collected 87 plant genomes with an estimated fraction of non-LTR retrotransposons. Among them, 11 (13%) harbor over 6% of non-LTR elements (range from 6% to 22%), and 17 (20%) contain less than 0.85%. As a result, the amplification of LINE elements in poplar is limited compared to the majority of other plants."

5. The choice of Populus simonii to confirm Celine in poplar centromeres is not adequately justified. The resolution provided by FISH and immunofluorescence signals may not substantiate the occupancy of CENH3 in these LINE sequences. Furthermore, the probes derived from poplar might exhibit deviations in signal intensity, and may not accurately reflect the signal strength differences in Populus due to the considerable variance in centromere sequences among different species.

Response: Ideally, pachytene chromosome-based cytological analyses should be done using a male plant of *P. trichocarpa.* Unfortunately, *P. trichocarpa* plants are rare in the Jiangsu Province and we were not able to find a male tree in Nanjing for cytogenetic analysis. As we indicate above, the CENH3-binding domain occupies approximately 1/50 of a pachytene chromosome. Thus, pachytene chromosome-based assays provide a very powerful tool to reveal whether a DNA element is associated and enriched within the functional centromere. In fact, most similar cytological assays were based on somatic metaphase chromosomes (Gong et al., 2012; Zhang et al., 2014; Nagaki et al., 2015; Han et al., 2016; Zhu et al., 2016; Li et al., 2018; Yang et al., 2018; Su et al., 2019; Huang et al., 2021; Liu et al., 2023), which has a significantly lower resolution because pachytene chromosomes are 15-50 times longer than the somatic metaphase chromosomes in the same plant species.

The DNA sequences of *Celine* from *P. trichocarpa* are highly homologous to those in *P. simonii*. Pt45 sequence shares 82%-98% similarity to the *Celine* elements in *P. simonii*. In our experience, a FISH probe with >70% sequence homology will not generate unambiguous difference on signal intensity on targets with 70-100% sequence homology. Therefore, we do not anticipate technical problems in application of our FISH probes on *P. simonii* chromosomes.

6. P7. Line 193. "These three centromeres are likely to be mostly composed of highly repetitive satellite repeats." needs further substantiation. No satellite repeats have been identified and reported in the enriched cluster repeats. FISH signals of Pt45 were detected in all the chromosomes, however, no Celine repeats were identified in the reference genome, while many

CRM repeats were assembled in the genome. It's very strange.

Response: We are sorry for this rather vague statement. The CENH3 ChIP-seq reads were mapped to the new reference genome (v4) of *P. trichocarpa*. A CENH3-binding domain was detected in 17 of the 19 *P. trichocarpa* chromosomes, except for Chr13 and Chr14 (**Figure S1**) (Note: a CENH3-binding domain associated with Chr3 was identified in the v4 assembly but not in the old assembly). These results suggest that the centromeres of these two chromosomes are likely composed of long arrays of satellite repeats, which are very difficult to be fully sequenced and assembled even with the long-read sequencing technologies. We have previously demonstrated a very similar case in potato (Gong et al., 2012). We now identified the satellite repeats associated with chromosomes 13 and 14 (**Figure S2**), which confirm the lack of these satellite repeats in the current reference genome. We have modified the relevant text to better explain this matter.

7. P7 line 202. "Thus, the total amount of centromeric sequences from the 19 P. simonii chromosomes 203 was estimated to be 13.11 {plus minus} 3.8 Mb." The estimation of centromere size using FISH signals of Pt45 and PL2 may not provide an accurate representation of the core CENH3 region. This method might not be suitable given the current advancements in genome sequencing and assembly.

Response: We respect the comment from the Reviewer. Both CENH3 mapping and fiber-FISH methods have pros and cons in determining the size of the centromeres, but combining these two methods provide the most comprehensive view. The accuracy of CENH3 mapping would depend on the quality of genome assembly with highly repetitive centromeric DNA sequences, as well as the quality of the anti-CENH3 antibody for immunoprecipitation. On the other hand, fiber-FISH is especially powerful for physical mapping of large genomic regions containing repetitive DNA sequences. Fiber-FISH can achieve a respectful resolution for such complex genomic regions. For example, a recent paper (https://pubmed.ncbi.nlm.nih.gov/35446419/) showed that the well-known mitochondrial DNA (mtDNA) insertion in chromosome 2 of *Arabidopsis thaliana* is 641 kb long. Our lab measured this mtDNA insertion by fiber-FISH in 2001 (20 years ago) as “618 plus/minus 42 kb” (https://www.pnas.org/doi/abs/10.1073/pnas.091110398), which is remarkably close to the current sequencing data.

8. P8. "Celine primarily targets AAGA/AATA or its variants as the 5' nicking site. In contrast, the target sequences of CRM elements contain few AT-rich sites, hence the two elements have distinct specificity at the sequence level." The current genome is published in 2006, and the results may not be accurate. The dissimilar targeting mechanisms between Celine and CRM, being different types of retrotransposons, are not surprising and warrant a more detailed exploration of the underlying mechanisms.

Response: As stated above, we have re-performed the analyses with the latest assembly and the distinct target specificity still holds. We agree the difference is not surprising, but it is worth to point out, and we would like to explore the underlying mechanism if we can secure the appropriate resources in the future.

9. P9 line 244. "presence of Celine elements in our recently developed reference genome of P.simonii, which was sequenced using Oxford Nanopore long-read methodology." However, no detailed information or citation were include in this manuscript. If this data is unpublished or if the genome is under restriction, its inclusion in this manuscript is inappropriate.

Response: The reference genome of *P. simonii* has been released and is indicated in the "Data availability" statement of the manuscript: The genome assembly of *P. simonii* has been deposited to Genome Warehouse in The National Genomics Data Center (NGDC) under accession number GWHDUDC00000000 (https://ngdc.cncb.ac.cn/gwh/).

Response to comments from Reviewer #2

This manuscript entitled "Celine, a LINE retrotransposon colonized in the centromeres of poplar chromosomes" by Xin et al. investigated a centromeric LINE element, Celine, in Populus species. Overall, the study is neat and straight forward. I have some questions.

1. Figure 2b, lines 217-219 and other places in the paper demonstrate that Celine elements are inserted in CRM LTR retrotransposons. It is not clear to me whether the amplification of Celine is due to its own activity or the byproduct of the amplification of the CRM elements. If the authors compare the ages of these CRM elements in centromeres, what is their amplification time? Is it similar to the divergence time of the Celine elements?

Response: We thank you for a great question. We knew that the insertions of CRM and *Celine* are independent, but we should have made it clear in the text. In the new **Figure 5** of the revised manuscript, we have compared the ages of the two elements side by side (see below). The average ages of them are comparable but the amplification spectrums are very different. Most *Celine* elements are amplified within 0.05 to 1 million years, whereas CRM seems to have continuous activity. Please also see our answer to question 7, which is a related question.

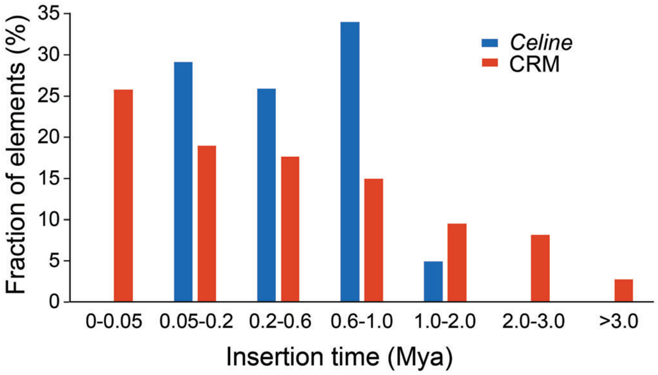

Figure 5. Estimated insertion time of full-length *Celine* elements and intact CRM elements in *P. trichocarpa*.

2. I am also curious whether the authors identified any centromeric satellite repeats associated with or near those Celine elements?

Response: We have identified a number of centromeric satellite repeats (see our response to minor comment #1 from Reviewer 1). Most of these repeats were derived or amplified from CRM elements (Table S1). However, amplification of CRM-related centromeric repeats has been reported in several plant species. Thus, we didn't want to include the results of these repeats in the manuscript, which is distractive to our focus on *Celine*. In the revised manuscript we have added a table listing the top 9 most abundant centromeric repeats identified in *P. trichocarpa* (Table S1) and FISH mapping of two satellite repeats associated with centromeres of chromosomes 13 and 14.

3. Abstract, Page 2, line 42, please also add the full name of "CRM" when it is first used.

Response: We have spelled out CRM.

4. Figure 1b, it seems that the red signal of the rightmost chromosome is not in the middle? Why?

Response: This chromosome is one copy of chromosome 10, which is an acrocentric chromosome in poplar karyotype. The short arm of chromosome 10 can not be consistently visualized on highly condensed somatic metaphase chromosomes (Xin et al., 2020).

5. Figure 2b, what are the red rectangles?

Response: The red boxes (rectangles) represent other classes of LTR retrotransposons, which is explained in the figure legend.

6. Results, page 6, line 160, please change into "in the P. trichocarpa genome".

Response: text has been modified.

7. Results, page 9, line 253-255, "Similar to what is observed in P. trichocarpa, most Celine elements (~65%) in P. simonii inserted into LTR retrotransposons, and some of them (~7%) were nested within themselves." I think it is really hard to tell whether there was one or multiple insertion events. It is largely possible that one or few Celine elements inserted into LTR retrotransposons, which got amplification, resulting in the amplification of the Celine elements. However, from the reading of here, it seems that the authors thought this was due to the multiple insertions. I do not see the data supporting it.

Response: This is a great question. We have addressed this comment in the revised text as below:

"The frequent insertion of *Celine* into other elements raises the question whether the amplification of *Celine* is due to the transposition of the target element carrying the *Celine*. If that is the case, one would expect duplicated TSD and flanking sequences among individual *Celine* elements. However, each *Celine* element has a unique TSD sequence (**Dataset 1** and **Dataset 2**). Moreover, all of the 14 CRM elements with identical LTRs do not contain *Celine* sequences or other nested insertions, suggesting elements with nested insertions are unlikely competent for further transposition. Those observations indicate that *Celine* amplifies through their own transposition machinery instead of piggybacking on other TEs including CRM elements."

8. Results, page 12, line 356-359, it seems to me that Celine became not active and less important for centromere function anymore.

Response: We agree with this comment from the Reviewer. Although *Celine* is more specific than CRM in targeting the functional centromeres marked by CENH3, *Celine*-like LINEs will eventually disappear from the genome due to its self-limiting amplification in a highly specialized chromosomal domain. Thus, any functional role of *Celine*-like LINEs will phase out gradually during evolution. Again, this may explain why *Celine*-like elements are discovered only in few phylogenetically unrelated eukaryotes. In contrast, CRM is a dominant centromeric DNA component in many plant species. For example, CRM has been found nearly universally among species in the grass family, Poaceae, which contains more than 10,000 species.

References

Gong ZY, Wu YF, Koblizkova A, Torres GA, Wang K, Iovene M, Neumann P, Zhang WL, Novak P, Buell CR, Macas J, Jiang JM (2012) Repeatless and repeat-based centromeres in potato: implications for centromere evolution. Plant Cell **24:** 3559-3574

Han JL, Masonbrink RE, Shan WB, Song FQ, Zhang JS, Yu WC, Wang KB, Wu YF, Tang HB, Wendel JF, Wang K (2016) Rapid proliferation and nucleolar organizer targeting centromeric retrotransposons in cotton. Plant Journal **88:** 992-1005

Huang YJ, Ding WJ, Zhang MQ, Han JL, Jing YNF, Yao W, Hasterok R, Wang ZH, Wang K (2021) The formation and evolution of centromeric satellite repeats in *Saccharum* species. Plant Journal **106:** 616-629

Li YJ, Zuo S, Zhang ZL, Li ZJ, Han JL, Chu ZQ, Hasterok R, Wang K (2018) Centromeric DNA characterization in the model grass *Brachypodium distachyon* provides insights on the evolution of the genus. Plant Journal **93:** 1088-1101

Liu Y, Yi CY, Fan CL, Liu Q, Liu SL, Shen LS, Zhang KB, Huang YH, Liu C, Wang YX, Tian ZX, Han FP (2023) Pan- centromere reveals widespread centromere repositioning of soybean genomes. Proceedings of the National Academy of Sciences of the United States of America **120:** e2310177120

Nagaki K, Tanaka K, Yamaji N, Kobayashi H, Murata M (2015) Sunflower centromeres consist of a centromere-specific LINE and a chromosome-specific tandem repeat. Frontiers in Plant Science **6:** 912

Su HD, Liu YL, Liu C, Shi QH, Huang YH, Han FP (2019) Centromere satellite repeats have undergone rapid changes in polyploid wheat subgenomes. Plant Cell **31:** 2035-2051

Xin HY, Zhang T, Wu YF, Zhang WL, Zhang PD, Xi ML, Jiang JM (2020) An extraordinarily stable karyotype of the woody *Populus* species revealed by chromosome painting. Plant Journal **101:** 253-264

Yang XM, Zhao HN, Zhang T, Zeng ZX, Zhang PD, Zhu B, Han YH, Braz GT, Casler MD, Schmutz J, Jiang JM (2018) Amplification and adaptation of centromeric repeats in polyploid switchgrass species. New Phytologist **218:** 1645-1657

Zhang HQ, Koblizkova A, Wang K, Gong ZY, Oliveira L, Torres GA, Wu YF, Zhang WL, Novak P, Buell CR, Macas J, Jiang JM (2014) Boom-bust turnovers of megabase-sized centromeric DNA in *Solanum* Species: rapid evolution of DNA sequences associated with centromeres. Plant Cell **26:** 1436-1447

Zhu ZX, Gui ST, Jin J, Yi R, Wu ZH, Qian Q, Ding Y (2016) The NnCenH3 protein and centromeric DNA sequence profiles of *Nelumbo nucifera* Gaertn. (sacred lotus) reveal the DNA structures and dynamics of centromeres in basal eudicots. Plant Journal **87:** 568-582

14-Mar-2024
Dr. Jiming Jiang
Michigan State University
East Lansing, Michigan 48824

MSID: PP2023-RA-01582R1
MS TITLE: Celine, a LINE retrotransposon colonized in the centromeres of poplar chromosomes

Dear Dr. Jiming Jiang:

I am pleased to inform you that your revised manuscript "Celine, a LINE retrotransposon colonized in the centromeres of poplar chromosomes" has been accepted for publication in Plant Physiology. Your manuscript will be checked for consistency with journal formatting and image standards by one of our Science Editors, who will contact you shortly. They may have a number of queries and suggestions for you. Please keep in mind that they work to increase the online visibility of your article and its attraction for readers from the widest of backgrounds. You will have the opportunity to review any revisions, and I hope you will work constructively with them to get the most out of your research. You will also have the opportunity to review all changes when you receive the author proofs for your article.

Once validated for publication, your article will be sent to a Science Editor for a final review of your manuscript and source files. After your final files are uploaded, your article will appear in the next available issue and the online Preview version of your article will be posted after your signed Copyright and Checklist forms and final source files are received.

Please be sure to include the following in your manuscript file:
Harvard style citations
List of all author contributions and funding information at the beginning of manuscript after the titles and author list.
List all supplemental materials associated with the article, with titles, at the end of the ms, before the references and acknowledgments section.
Complete Figure Legends for all figures appearing in the article listed before the Literature Cited.

Thank you and congratulations on your Plant Physiology paper!

Sincerely,
Board of Editors, Plant Physiology

=====
FIRST AUTHOR PROFILES
=====

We recognize the hard work that goes into being First Author of a Plant Physiology paper by publishing a "First Author Feature" for each First Author alongside your paper.

If the first author(s) would like to submit information for a feature on Facebook and Twitter, fill out the form by clicking on the link below:

Plant Physiology First Author Feature Form: https://forms.office.com/r/ZBCGZfhm8F
If you cannot access the form through the link above, please contact Rachel at rbelsky@aspb.org.

To upload an image of yourself to accompany your profile, please put your photo in the correct journal folder at the link below. You must label your image: Author First Name Last Name Article Title.

First Author Profile Image Dropbox: https://www.dropbox.com/scl/fo/xz029p7qjr2jml2rp2kmq/h?dl=0&rlkey=6fkxcaqmxx35ap152b2osy1fz

=====
IMPORTANT REMINDER: PEER REVIEW REPORTS
=====

If you opted to publish a peer review report along with your article during the original submission process, it will be prepared by the editorial staff and publicly posted with your manuscript, inside the zip file that contains any other supplemental material. As a reminder, the peer review report is a public record of all comments from editors and reviewers, as well as your prior responses, as you received them in the decision letters for each draft of your manuscript. If you agreed to publish this report and have changed your mind, or are not sure if you selected this option, please contact the editorial office as soon as possible before signing the license agreement from our publisher.

-- Reviewer comments:
Reviewer #1 (Comments for the Author):

The authors have addressed most of my comments. A remained concern is the the genome assembly of P. simonii that has been deposited to Genome Warehouse in The National Genomics Data Center (NGDC) under accession number GWHDUDC00000000. No supporting information or citation to confirm the quality and reliability of the assembled genome. Is the genome published?

Reviewer #2 (Comments for the Author):

The authors addressed the majority of my questions raised in the last version. This is a neat paper describing a centromere specific LINE element. However, it did not tell too much about the function of Celine, particularly how it has shaped the function of poplar centromeres. Furthermore, this LINE element seems not so active anymore, and maybe is losing their function in centromere evolution.
